# *In vitro* histone lysine methylation by NSD1, NSD2/MMSET/WHSC1 and NSD3/WHSC1L

**DOI:** 10.1186/s12900-014-0025-x

**Published:** 2014-12-12

**Authors:** Masayo Morishita, Damiaan Mevius, Eric di Luccio

**Affiliations:** Kyungpook National University, School of Applied Biosciences, Life Sciences and Agriculture building #3, room 309, 80 Daehak-ro, Daegu, Buk-gu 702-701 Republic of Korea

**Keywords:** Epigenetic therapy of cancer, Histone lysine methyltransferase, NSD1, NSD2/MMSET/WHSC1, NSD3/WHSC1L, HMTase inhibitors

## Abstract

**Background:**

Histone lysine methylation has a pivotal role in regulating the chromatin. Histone modifiers, including histone methyl transferases (HMTases), have clear roles in human carcinogenesis but the extent of their functions and regulation are not well understood. The NSD family of HMTases comprised of three members (NSD1, NSD2/MMSET/WHSC1, and NSD3/WHSC1L) are oncogenes aberrantly expressed in several cancers, suggesting their potential to serve as novel therapeutic targets. However, the substrate specificity of the NSDs and the molecular mechanism of histones H3 and H4 recognition and methylation have not yet been established.

**Results:**

Herein, we investigated the *in vitro* mechanisms of histones H3 and H4 recognition and modifications by the catalytic domain of NSD family members. In this study, we quantified *in vitro* mono-, di- and tri- methylations on H3K4, H3K9, H3K27, H3K36, H3K79, and H4K20 by the carboxyl terminal domain (CTD) of NSD1, NSD2 and NSD3, using histone as substrate. Next, we used a molecular modelling approach and docked 6-mer peptides H3K4 a.a. 1-7; H3K9 a.a. 5-11; H3K27 a.a. 23-29; H3K36 a.a. 32-38; H3K79 a.a. 75-81; H4K20 a.a. 16-22 with the catalytic domain of the NSDs to provide insight into lysine-marks recognition and methylation on histones H3 and H4.

**Conclusions:**

Our data highlight the versatility of NSD1, NSD2, and NSD3 for recognizing and methylating several histone lysine marks on histones H3 and H4. Our work provides a basis to design selective and specific NSDs inhibitors. We discuss the relevance of our findings for the development of NSD inhibitors amenable for novel chemotherapies.

**Electronic supplementary material:**

The online version of this article (doi:10.1186/s12900-014-0025-x) contains supplementary material, which is available to authorized users.

## Background

Covalent histone modifications are key in chromatin regulatory mechanisms. One such histone modification, lysine methylation, can have both activating and repressive functions on transcriptional events. Histone lysine methyltransferases (HMTases) are transcriptional co-regulators that target specific lysines on histones H3 and H4, and can transfer up to three methyl groups (Kme1, Kme2, and Kme3) [1]. However, the molecular mechanisms of histone mark recognition remain unclear [2-6]. Epigenetic marks on H3K4, H3K9, H3K27, H3K36, H3K79, and H4K20 have been reported to play primary roles in regulating the chromatin and contribute to the histone code that is still obscure [7]. H3K4, H3K36 and H3K79 methylation are associated with a locally relaxed chromatin and transcriptionally active genes, whereas methylation of H3K9, H3K27 and H4K20 are repressors and hallmarks of condensed chromatin at silent *loci*. Dysfunctions in the regulation of histone methylation are linked to an increasing number of pathological conditions.

The nuclear receptor-binding SET domain (NSD) family, a part of the HMTase KMT3 family, is composed of three HMTases: NSD1, NSD2/MMSET/WHSC1, and NSD3/WHSC1L1 (hereafter NSD1, NSD2 and NSD3) [8]. The NSD proteins are oncogenes highly expressed in numerous pathological conditions and are considered attractive novel therapeutic targets in cancers [2,9-20]. The amplification of NSD1 has been reported in multiple myeloma, lung cancer, neuroblastoma and glioblastoma. The amplification of either NSD1 or NSD2 triggers cellular transformation [21-28]. NSD2 is associated with tumor aggressiveness or prognosis in most types of cancers, including prostate cancer and multiple myeloma and is overexpressed in at least 15 different cancers [9,10,17,18,20,21,25,29-33]. Increased NSD2 activity is also reported during tumor proliferation in glioblastoma and myeloma [34], resulting in aberrantly high global levels of H3K36me2 [18]. NSD3 is amplified in primary breast carcinoma, bladder cancer, lung cancer, and liver cancer [12,16,25,35]. Abnormal fusion proteins containing NSD family members, including NSD1-NUP98 and NSD3-NUP98 fusions, increase H3K36 methylation, leading to acute myeloid leukemia [24-28].

Although NSD proteins are instrumental in the development of numerous cancers, their mechanisms of action in carcinogenesis are still unclear. In particular, the substrate specificity of the NSD members remains unsettled and conflicting data have been reported [17,20]. NSD1 has H3K36-specific mono- and di-methylation activities but methylation on H4K20 by NSD1 has also been reported [3,23,27,36]. NSD2 activity performs H3K36-specific mono- and di-methylation on chromatin, but di-methylation activity for H4K20 at DNA double-strand breaks has also been reported [17,20]. A significant increase in H3K36me3 by NSD2 at promoter regions has also recently been demonstrated [4], but potentially ruled-out in another study [20]. In addition, NSD2 has been shown to methylate H3K4, H3K27, and H4K44 [5,6,36-38]. Discrepancies regarding NSD3 methylation activities on H3K4, H3K27, and H3K36 have also been reported [6,24,39-41]. Complicating the mechanisms further, both NSD1 and NSD2 have been shown to methylate NF-κB [8,42]. Thus, no consensus on the substrate specificity of the NSD members has been established [17,20,36]. Multiple factors may be responsible for these discrepancies, such as the cellular context, the nature of the substrate (peptide, histone, or nucleosome), the integrity of the enzymes (catalytic domain alone versus full-length protein), and the assays employed.

Despite uncertainties regarding NSD substrates, it appears that increased levels of H3K36 methylation concomitant with decreased levels of H3K27 methylation by deregulated NSD members is a driving mechanism in chromatin regulation [13]. Therefore, the inhibition of NSD members by small molecules represents a valuable therapeutic strategy in several human cancers where elevated levels of NSD members have been reported [2,4,5,12,14,17,18,26,32,33,43-45].

**Figure 1:**
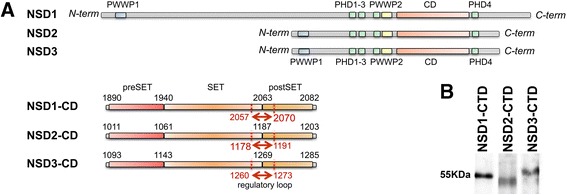
**The NSD family of HMTase. A**- The schematic primary structure of NSD1, NSD2, and NSD3: All NSD members contain two PWWP domains, four PHD zinc finger domains, and a CD. The CD has HMTase activity and contains the pre-SET, SET, and post-SET domains. The regulatory loop over the histone-binding site is indicated in red. **B**- Coomassie Blue staining of a SDS-PAGE gel confirms the expressed recombinant NSD1-CTD, NSD2-CTD, and NSD3-CTD.

The NSD family members are large multi-domain proteins with four zinc finger domains, two PWWP domains and a catalytic SET domain (Figure 1A) [9,10,17,20]. The SET domain is subdivided into pre-SET, SET, and post-SET domains, all domains are required for catalytic function (Figure 1A). The function of PHD1-3 and PWWP1-2 domains of the three NSD members on chromatin remains elusive. However, a recent study demonstrated the role of the conserved PHD4 domain in localizing NSD3 on histone H3 [46]. The PHD4 domain of the NSDs may function as a recognition module for histone-lysine marks, helping to position the catalytic SET domain on its lysine substrate [46]. The PHD4 domain is 36-residues downstream of the post-SET subdomain. Due to its immediate vicinity to the post-SET subdomain, PHD4 may be a structural component of the catalytic SET domain. Similarly, the PWWP2 domain is also conserved across NSD members and 72-residues upstream of the pre-SET subdomain. The role of this PWWP2 domain remains unclear.

Previous studies have investigated either full-length NSD members or the catalytic SET domain alone through various experimental approaches, resulting in discrepant conclusions. Herein, we focus on understanding histones H3 and H4 recognition and methylation *in vitro* by the NSDs. In this study, we investigate the function of the carboxyl terminal domain (CTD) of all three NSD members using a consistent experimental approach, *in vitro*. The NSDs-CTD contains the pre-SET, SET, post-SET, and PHD4 domains within the catalytic core as well as a histone-recognition module. We quantified the pan-methylation properties of the CTD of NSD1, NSD2, and NSD3 on H3K4, H3K9, H3K27, H3K36, H3K79, and H4K20 *in vitro*. Next, we used MD simulations to elucidate the binding of the NSD catalytic SET domain (CD) on histone tails. Taken together, this study provides insights relevant to the design of specific and selective NSD inhibitors.

## Methods

### Cloning

The carboxyl terminal domains (CTD) of *NSD1*, *NSD2*, and *NSD3* genes were cloned as follows; *NSD1-CTD* (1338 bp, 5325–6663 nt; encoding 446 a.a., 1775–2221 a.a.), *NSD2-CTD* (1287 bp, 2808–4095 nt; encoding 429 a.a., 936–1365 a.a.), and *NSD3-CTD* (1344 bp, 2967–4311 nt; encoding 484 a.a., 989–1437 a.a.) were amplified by PCR using a human liver cDNA library (TAKARA, Japan) as the template. The sets of forward and reverse primers used were 5′-GCTGAG*GTCGAC*(*Sal*I)CATCCTCGAGCTGTTCCTTCC-3′ and 5′-CGGTAC*GCGGCCGC*(*Not*I)GCACATACTCACGGATCTCCCC-3′ for *NSD1-CTD*, 5′- CAGGCG*CATATG*(*Nde*I)TTCCCGTACATGGAGGGGGAC-3′ and 5′- CGCCTG*CTCGAG*(*Xho*I)TTTGCCCTCTGTGACTCTCCG-3′ for *NSD2-CTD*, and 5′- CTGAAC*GTCGAC*(*Sal*I)GGCCTTAAACATGACTTGGGG-3′ and 5′- GACACC*GCGGCCGC*(*Not*I)ATTCTTTTACTTCTTCTCCATG-3′ for *NSD3-CTD*. The PCR-amplified *NSD1-CTD* and *NSD3-CTD* fragments (with *Sal*I and *Not*I restriction sites) and the *NSD2-CTD* fragment (with *Nde*I and *Xho*I restriction sites) were inserted into the corresponding restriction enzyme sites of the multi-cloning site of the protein expression intein-tagging vectors pTYB2 or pTYB12 (New England Biolabs, USA). All sequences were verified by sequencing.

### Protein expression and purification

*Escherichia coli* expression BL21 (DE3) strain was transformed with pTYB2 or pTYB12 plasmids harbouring *NSD1-CTD*, *NSD2-CTD*, and *NSD3-CTD* (*NSD-CTDs*). The transformed strains were grown in LB medium containing 100 μg/mL ampicillin and the expression of recombinant NSD-CTDs was induced with 250 μM isopropyl 1-thio-D-galactopyranoside (IPTG) for 4 h at 15 °C. *E. coli* cells were harvested and lysed by a freeze-thaw method and incubated in buffer A [20 mM Tris (pH 8.5), 500 mM NaCl and 0.1 mM EDTA] containing 0.1 % Triton X-100 and 1 mM phenylmethanesulfonylfluoride (PMSF) and treated with 20 cycles of sonication on ice. The resulting cell extract containing NSD-CTD-Intein-CBD (chitin-binding domain) fusion proteins was loaded onto an affinity column of chitin beads and washed with 100 column volumes of buffer A with 0.1 % Triton X-100, followed by 20 column volumes of buffer A without Triton X-100. To remove bacterial chaperones bound to the recombinant proteins, the recombinant NSD-CTD-bound chitin beads were washed with 10 bed volumes of buffer A containing 10 mM adenosine triphosphate (ATP) and 2.5 mM MgCl_2_. The affinity column was washed with 20 bed volumes of buffer A and NSD-CTD proteins were cleaved off from the chitin beads by incubation in buffer A with 50 mM 2-mercaptoethanol at 4 °C for 64 h. After elution in buffer A, samples were concentrated and 2-mercaptoethanol was washed off using Amicon Ultra centrifugal filters (Millipore, USA) and then used for methyltransferase assays. A small portion of purified NSD-CTDs was resolved through SDS-PAGE and Coomassie staining showed soluble and pure NSD-CTDs at approximately the expected molecular weight of 52.9 kDa (NSD1-CTD), 49.7 kDa (NSD2-CTD) and 53.1 kDa (NSD3-CTD) (Figure 1B). The resulting recombinant NSD-CTDs proved stable and retained their catalytic properties for an extended period of time when stored at −80 °C.

### Histone methyltransferase assays

**Figure 2:**
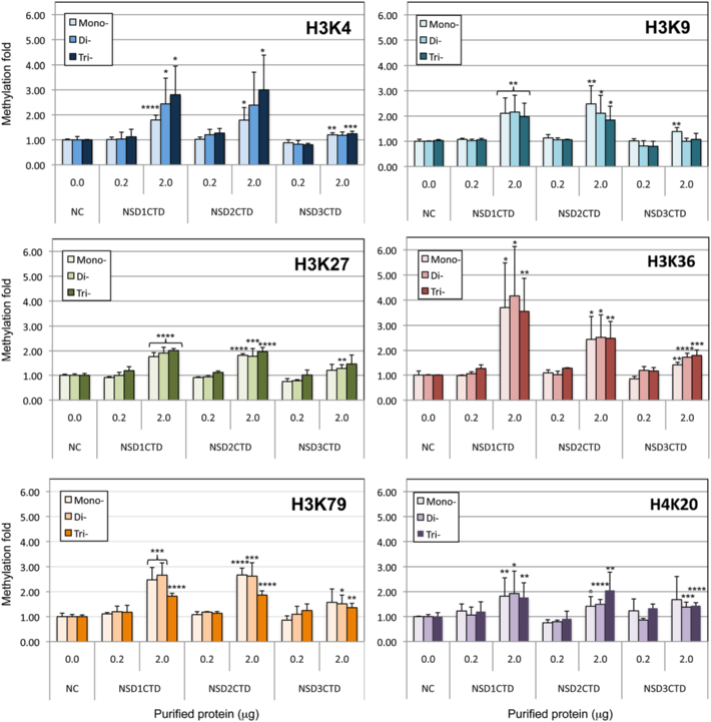
**HMTase activities of NSD1-CTD, NSD2-CTD, and NSD3-CTD on H3 and H4 substrates** ***in vitro.*** HMTase activities of NSD1-CTD, NSD2-CTD, and NSD3-CTD on H3K4, K9, K27, K36, K79, and H4K20 were measured using colorimetric quantification (See Materials and Methods). HMTase assays were done in duplicate individual experiments and all samples were in duplicate. The error bars indicate standard deviations. The results were normalized against controls that did not contain any enzymes (NC, 0 μg). Asterisks indicate P-values; *p < 0.05, **p < 0.02, ***p < 0.005, ****p < 0.001.

Histone methyltransferase activities of NSD-CTDs on H3K4, K9, K27, K36, K79, and H4K20 were measured using colorimetric quantification kits (Epigentek, USA) following the manufacturer’s protocol. As for H3K36, H3K79 and H4K20, the purified recombinant NSD-CTDs (0.2 μg and 2.0 μg) were incubated with a recombinant histone H3 or H4 (Epigentek, USA) and a methyl group donor (SAM) in the strip wells coated with the specific antibodies for 60–90 min at room temperature. The specific antibodies attached to the bottom of the strip wells captured methylated substrates. As for H3K4, H3K9, and H3K27, instead of the antibody-coated strip wells, biotinylated histone substrates were used and stably captured on the strip wells during the 60 min incubation at 37 °C. The captured biotinylated histone substrates were next incubated with a high-affinity antibody for 60 min at room temperature with shaking at 100 rpm. Excess of purified NSD-CTDs, histones, SAM, and antibodies were thoroughly washed away and the strip wells attached with antibody-bound or biotinylated H3/H4me were incubated with the labeled detection antibodies for 60 min at 24 °C, swirling at 100 rpm in the dark. After thoroughly washing the wells, the methylation level was quantified through a HRP-conjugated secondary antibody-color development system with an ELISA plate reader at 450 nm. The antibodies provided by Epigentek are validated and showed no cross-reaction between the substrates. Assays were performed in duplicate. The results were normalized against a control that did not contain any enzymes (NC in Figure 2).

### Molecular modeling, docking, and energy minimization

The crystal structure of the catalytic domain (CD) of NSD1 (PDB: 3OOI - a.a. 1850–2080) served as a template for building homology models of NSD2-CD (a.a. 971–1202) and NSD3-CD (a.a. 1044–1283). Multiple-sequence alignment was done with ClustalW V2.0.9 and 100 models were generated using Modeler V9.5. The best model, according to the intrinsic Modeler function, was subjected to side-chains positioning using the SCWRL4 program. Stereochemistry was checked with PROCHECK. The model was manually inspected with COOT. The histone tail peptides were manually built in COOT with the following residues: H3K4 (a.a. 1–7), H3K9 (a.a. 5–11), H3K27 (a.a. 23–29), H3K36 (a.a. 32–38), H3K79 (a.a. 75–81), and H4K20 (a.a. 16–22). A structural overlay between NSD1-CD (PDB: 3OOI) and the crystal structure of SET8 bound to histone H4 peptide (PDB: 3F9Y) was used to manually place and orientate the H3- or H4-peptide tails into the histone binding site of NSD1-CD, NSD2-CD, and NSD3-CD using the SSM algorithm in COOT. Prior to MD calculations, all the raw modelled histone tail peptides exhibited a similar conformation. Electrostatic charge calculations were performed using the PDB2PQR Server (http://nbcr-222.ucsd.edu/pdb2pqr_1.9.0/) with default settings (PARSE forcefield) regardless of the conformation of the peptide tail. We hypothesized the electrostatic charge calculations were not affected by the conformation of the peptide tail as all modelled short peptide tails have a highly similar initial conformation.

Electrostatic calculations were performed with APBS V1.2.1 and the molecular surfaces with the electrostatic properties were rendered with both VMD V1.8.7 and PyMOL V1.2.

### Energy minimizations and molecular dynamics simulations in a water box

The peptide-NSD-CD complexes were fully solvated in a water box (140 Å × 100 Å × 110 Å) using VMD 1.9. The molecular systems used for energy minimizations (EM) and molecular dynamics (MD) contained the following number of atoms: NSD1-CD-H3K4(1–7), 42,121 atoms; NSD1-CD-H3K9(5–11), 42,104 atoms; NSD1-CD-H3K27(23–27), 42,095 atoms; NSD1-CD-H3K36(32–38), 42,106 atoms; NSD1-CD-H3K79(75–81), 42,120 atoms; NSD1-CD-H4K20(32–38), 42,130 atoms; NSD2-CD-H3K4(1–7), 44,848 atoms; NSD2-CD-H3K9(5–11), 44,840 atoms; NSD2-CD-H3K27(23–27), 44,828 atoms; NSD2-CD-H3K36(32–38), 44,830 atoms; NSD2-CD-H3K79(75–81), 44,844 atoms; NSD2-CD-H4K20(32–38), 44,860 atoms; NSD3-CD-H3K4(1–7), 44,367 atoms; NSD3-CD-H3K9(5–11), 44,353 atoms; NSD3-CD-H3K27(23–27), 44,341 atoms; NSD3-CD-H3K36(32–38), 44,349 atoms; NSD3-CD-H3K79(75–81), 44,366 atoms; NSD3-CD-H4K20(32–38), 44,379 atoms. About 90 % of the atoms were from the water molecules. EM and MD were performed with NAMD v2.8 using the CHARMM force field. A constant temperature was maintained using a Langevin damping at 300 K, and the pressure was held constant at 1 atmosphere with a Langevin piston. Electrostatic forces were taken into account using the particle mesh Ewald (PME) method with a 10 Å cut-off distance. The solvated complexes, histone-peptide-NSD-CD, were first equilibrated with 5,000 steps (5 ns) of conjugate gradient EM followed by 5 cycles of 20,000 steps (20 ns) of MD and 5,000 steps (5 ns) of EM. The last step was 5 ns of EM. Each solvated complex of histone-peptide-NSD-CD was subjected to a total of 100,000 steps (100 ns) of MD and a total of 30,000 steps (30 ns) of EM. EM steps were long enough to reach a local minimum with Δε < 1 kJ/mol. After the MD simulations, the solvated complexes were manually checked for ideal stereochemistry with COOT. The MD trajectories were analyzed with VMD 1.9 and the plots of the total energy *versus* timescale were plotted using Gnuplot 4.4.

### Binding energy determination

Binding energy was calculated as follows: ε_binding_ = ε_complex_ – (ε_peptide_ + ε_NSD_) using the total energy calculations of NAMD [47]. Models of the last MD step were stripped from solvent molecules and used to calculate the binding energy of the complex. Models of the binding complex, peptides, and NSD-CD apo were all subjected to the same EM procedure prior to binding energy calculation. Models were energy minimized with 4,000 steps (4 ns) of conjugate gradient EM followed by 2,000 steps (2 ns) of MD and 6,000 steps (6 ns) of EM (NAMD v2.8) [47]. EM steps were long enough to reach a local minimum with Δε < 1 kJ/mol. A constant temperature was maintained by using Langevin damping at 300 K and the pressure was held constant at 1 atmosphere with a Langevin piston.

### Model analysis and validation

The details of the interactions between the NSD-CD and H3- or H4- peptides were analyzed with Ligplot [48]. The confirmation of interaction maps was manually done through visual inspections of the models in COOT [49].

## Results

### The CTD of NSD members can methylate multiple histone lysines *in vitro*

To examine whether the CTD of NSD1, NSD2, and NSD3 specifically recognizes and methylates multiple lysines of H3 and H4 *in vitro*, we quantified the pan-methylation of NSD1-CTD, NSD2-CTD, and NSD3-CTD on H3K4, H3K9, H3K27, H3K36, H3K79, and H4K20 (Figure 2).

Reinberg and colleagues reported that NSD2-SET domain methylates H4K44 on octamers, which could be linked to histone deposition and/or to the response to DNA damage [36]. Although H4K44 methylation remains to be investigated, it was omitted in this study due to lack of a suitable and consistent assay.

Overall, NSD1-CTD, NSD2-CTD, and NSD3-CTD exhibit similar substrate recognition and methylation properties, but reduced methylation activities were observed for NSD3-CTD (Figure 2). H3K4me3 and H3K27me3 species were preferably detected rather than –me1 and –me2 species for both NSD1-CTD and NSD2-CTD. In contrast, H3K9-me1 & -me2 and H3K79-me1 & -me2 species were favored against –me3 species.

Unlike NSD1-CTD and NSD2-CTD, NSD3-CTD showed a lower, but significant, activity (up to ~1.5 fold increase in methylation fold for almost all substrates tested, except for the marginal activity on H3K4 and H3K9) and the methylation properties of NSD3-CTD on H3K9, H3K27, and H3K79 were almost identical to those of NSD1-CTD and NSD2-CTD. However, H4K20 methylation varied among all NSD-CTDs. Nonetheless, significant activity of NSD1-CTD on mono-/tri-methylation, NSD2-CTD on di-/tri-methylation, and NSD3-CTD on di-/tri-methylation of H4K20 were observed, indicating that H4K20 is an *in vitro* target for all NSD-CTDs. Importantly, H3K36 was methylated by all NSD-CTDs and appears to be the preferable *in vitro* substrate for NSD1-CTD.

Taken together, our data indicate that, *in vitro,* the CTD of NSD1, NSD2, and NSD3 is able to recognize and methylate H3K4, H3K9, H3K27, H3K36, H3K79, and H4K20 with significant differences in catalytic activities depending on the substrate. Although some HMTases (such as SET9) do not retain significant methyltransferase activity *in vitro* [50], this was not the case for NSD members, where significant enzymatic activities were measured (Figure 2).

### Molecular modelling and the open-and-closed conformation of the catalytic domain

**Figure 3:**
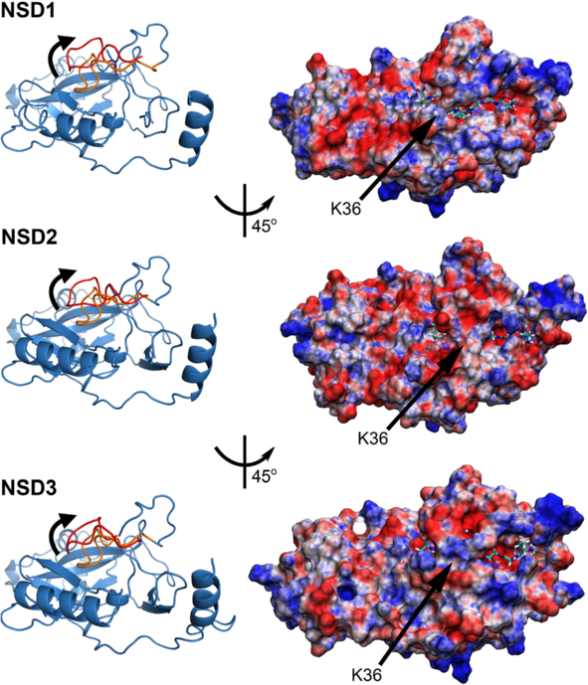
**The opening motion of the catalytic domain. **Left panel: The structure of the CD is composed of three groups of canonical β-sheets arranged in a triangular fashion with a group of two β-sheets closely neighbouring a conserved α-helix defining a cleft for the binding of the lysine-histone ligand. The cofactor SAM binds in a cavity adjacent to the CD connected through a channel. The flexible regulatory loop is shown in red. The motion of the regulatory loop is indicated with bent black closed arrows. Right panel: Model of the NSD-CDs bound with the H3-peptide (a.a. 32–38) after MD simulations. H3K36 is indicated by an arrow. The electrostatic surface is colored as follows: blue: positive charges; red: negative charges with unit +5/-5 kb.T.e_c_^−1^.

Our data showed that, *in vitro,* the CTD of NSD1, NSD2, and NSD3 has the ability to recognize and methylate several histone lysines (Figure 2). To gain further insight into histone lysine methylation by the NSD members, we investigated conformational modifications of the catalytic domain (CD) to gain access to the lysine-binding pocket. The crystal structure of NSD1-CD (PDB: 3OOI) is devoid of substrate and the histone-lysine binding area is occluded by a regulatory loop at the interface of the SET and post-SET regions (Figure 3) [9,51]. Previously, we showed that molecular determinants of this regulatory loop extend over the histone-binding site, sterically preventing substrates from binding [51-53]. The H3K4 HMTase MLL1 also has been described to have a flexible post-SET responsible for the open-and-closed conformation of the lysine-substrate binding site [53].

The crystal structure of NSD1-CD (PDB: 3OOI - a.a. 1850–2080) was used to build the corresponding CDs of NSD2 and NSD3 by homology modelling. A pair-wise sequence alignment between the template and the target showed that the NSD2-CD (a.a. 971–1202) shares 75.9 % identity and 90.1 % similarity with the NSD1-CD (PDB: 3OO1), whereas the NSD3-CD (a.a. 1044–1283) shares 72.9 % identity and 85 % similarity with the NSD1-CD. Protein sequences sharing at least 40 % identity are likely to share similar structures, thus all NSD1-CD, NSD2-CD, and NSD3-CD are likely to share a similar scaffold (Figure 1).

**Figure 4:**
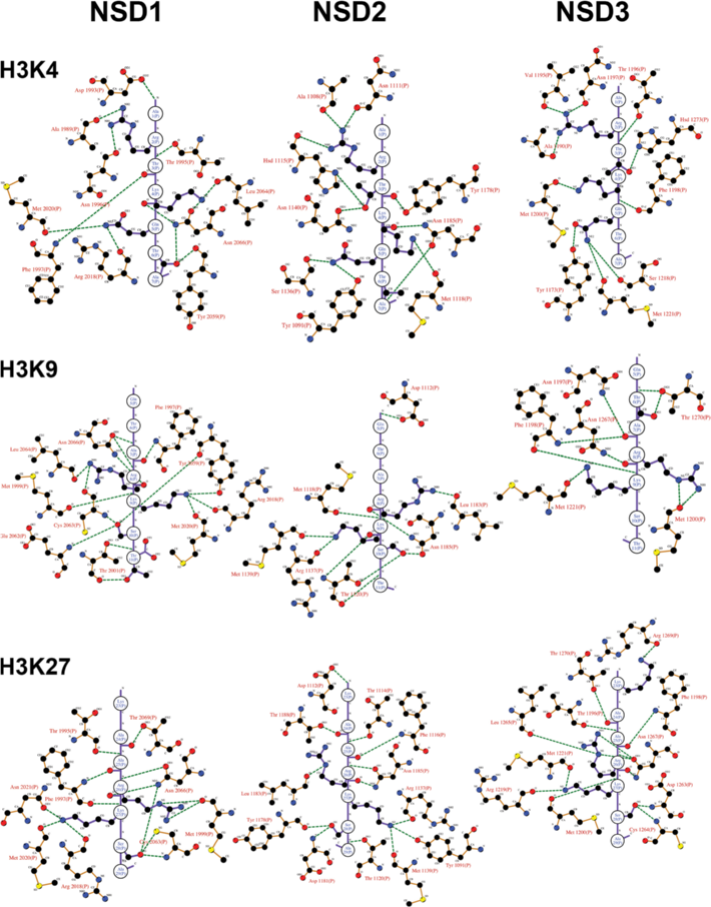
**Molecular determinants of the binding of H3K4, H3K9 and H3K27.** The peptides H3K4 (a.a. 1-7), H3K9 (a.a. 5–11), and H3K27 (a.a. 23–29) are oriented vertically and depicted in purple with the atomic details of the side chains. The interacting amino acids from the CDs of the NSD members are indicated in brown and numbered. Green dashed lines represent the hydrogen bonds. The hydrophobic interactions are omitted to declutter the figure. The schematic was generated with the program Ligplot.

**Figure 5:**
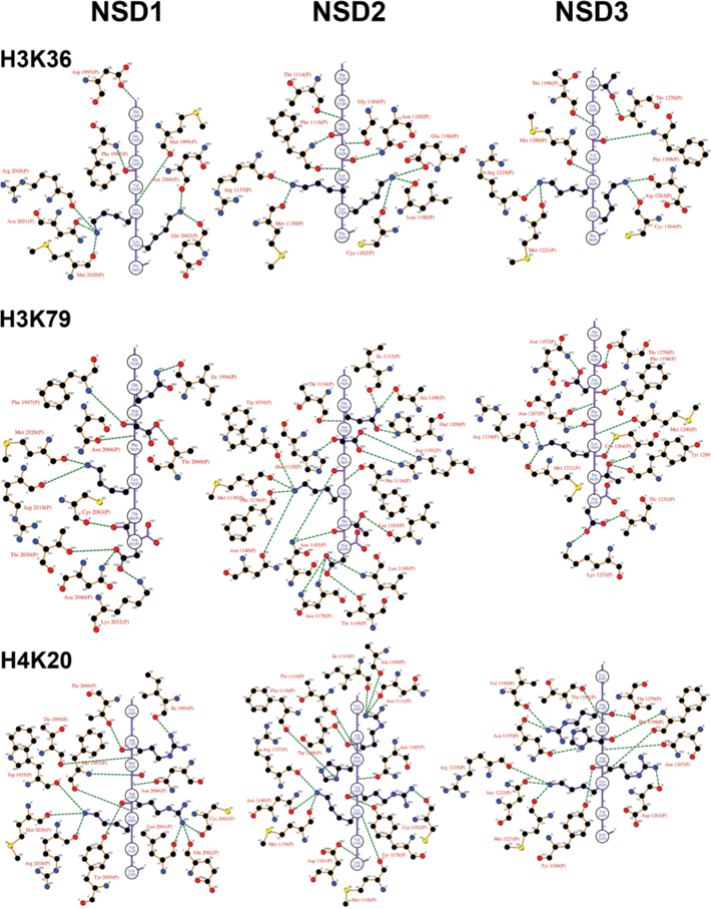
**Molecular determinants of the binding of H3K36, H3K79 and H4K20.** The peptides H3K36 (a.a. 32–38), H3K79 (a.a. 75–81), and H4K20 (a.a. 16-22) are shown. Details are the same as in Figure 4.

Next, we used molecular dynamics (MD) to investigate the docking of peptides containing histone lysine (H3K4, H3K9, H3K27, H3K36, H3K79, and H4K20), NSD1-CD, NSD2-CD, and NSD3-CD. The initial models were based on the crystal structure of NSD1-CD solved in a closed apo-conformation. Therefore, models of the apo CD of NSD1, NSD2, and NSD3 docked with peptides were deliberately brought into a sterically unstable conformation. Energy minimizations and long MD simulations performed in a water box centered on the protein relieved the steric clashes and forced the CDs into an open conformation that accommodates substrates (Figures 3, 4, 5 and Additional file 1: Figure S1). Long-range MD simulations were applied to allow complexes to equilibrate into stable conformations with little RMSD oscillations observed for the post-SET regions (overall c_α_-RMSD < 0.6 Å) [9]. The resulting models may approximate the conformations of the opened CD potentially resulting from the binding of nucleosomal DNA as an allosteric effector. Following the MD simulations, the structural modifications observed were on the CD of NSD2 and NSD3, specifically on a loop at the interface between the SET and post-SET regions (a.a. 1178 to 1191 for NSD2-CD and a.a. 1260–1273 for NSD3-CD). This specific loop rotated ~40° and translated ~6 Å at the tip, which is a significant displacement (Figure 3) [9]. The rest of the backbone did not undergo significant structural modifications, with an overall c_α_-RMSD < 0.6 Å.

### Molecular binding details of H3K4, H3K9, H3K27, H3K36, H3K79, and H4K20 peptides

The CDs of the NSDs are likely to share highly similar structural features (Figure 3). The histone tail-binding area only becomes accessible when the regulatory loop at the interface between the SET and post-SET regions is rotated and displaced, exposing a negatively-charged surface area suitable for the docking of the positively charged H3 and H4 peptide tails (Figure 3).

As a step forward towards understanding the molecular details of substrate recognition by NSD-CDs, we used MD simulations to examine the binding characteristics of H3K4, H3K9, H3K27, H3K36, H3K79, and H4K20 peptides. Overall, our data indicate that all the histone H3 and H4 peptides tested (H3K4 a.a. 1-7; H3K9 a.a. 5-11; H3K27 a.a. 23-29; H3K36 a.a. 32-38; H3K79 a.a. 75-81; H4K20 a.a. 16-22) share similar steric hindrance and have similar positive charges related to their electric field and molecular surface. These properties imply favourable binding energies for the NSD-CDs (Table 1).

**Table 1:** **Binding energy of peptides containing lysine-histone substrates onto the CD of NSD1, NSD2, and NSD3**

**(kcal/mol)**	**NSD1**	**NSD2**	**NSD3**
**H3K4**	−229.7	−299.4	−200.5
**H3K9**	−353.2	−280.4	−282.9
**H3K27**	−151.9	−252.6	−247.4
**H3K36**	−277.1	−272.2	−261.6
**H3K79**	−285.6	−332.4	−254.3
**H4K20**	−277.2	−269.5	−289.0
**polyE**	444.2	862.9	663.2

Peptides are as follows: H3K4 (a.a. 1–7); H3K9 (a.a. 5–11); H3K27 (a.a. 23–29); H3K36 (a.a. 32–38); H3K79 (a.a. 75–81); H4K20 (a.a. 16–22). The polyE is a 6-mer poly-glutamic acid peptide used as a control experiment for the docking studies.

The total energy of the complexes after the MD simulations was highly comparable between the tested substrates (standard deviation of 0.12 % for both NSD1 and NSD2, and 0.11 % for NSD3), indicating that all peptides tested similarly bind to the NSD members (Table 1). In addition, the regulatory loop acts as a “seat belt” to anchor a single histone tail into the binding domain, which allows the lysine substrate to extend toward the cofactor-binding site for the catalytic reaction to occur (Figure 3). The peptide-binding area is large enough to accommodate a seven-amino acid peptide. Interestingly, the seven-a.a. peptides (containing H3K4, H3K9, H3K27, H3K36, H3K79, and H4K20) share highly similar steric and electrostatic properties (Figures 3, 4 and 5). In addition, all the H3 and H4 histone peptides tested were also firmly stabilized into the histone-tail binding cleft of NSD1, NSD2, and NSD3 by an extensive network of hydrogen bonds (Figures 4 and 5). This observation is consistent with the calculated binding energies, reflecting the proper steric and electrostatic compatibilities between histone peptides and the CDs (Table 1).

The binding of histone H3 and H4 peptides induces significant modifications to the histone-binding pocket. In addition to the movement of the regulatory loop seating on top of the histone tail, an induce-fit mechanism triggers changes locally in the backbone along with the rotation of the side chain of key amino acids creating an extensive network of hydrogen bonds anchoring the H3 and H4 peptides (Figures 4 and 5). Specifically, H3K4 (a.a. 1-7) peptide is stabilized by hydrogen bonds with Ala1989, Asp1993, Thr1995, Asn1996, Phe1997, Met2020, Arg2018, Tyr2059, Leu2064 and Asn2066 in NSD1, with Tyr1091, Ala1108, Asn1111, His1115, Met1118, Ser1136, Asn1140, Tyr1178 and Asn1185 in NSD2, and with Tyr1173, Ala1190, Val1195, Thr1196, Asn1197, Phe1198, Met1200, Ser1218, Met1221, and His1273 In NSD3. H3K9 (a.a. 5-11) peptide is stabilized by hydrogen bonds with Phe1997, Thr2001, Arg2018, Met2020, Met1999, Tyr2059, Glu2062, Cys2063, Leu2064 and Asn2066 in NSD1, with Asp1112, Met1118, Thr1120, Arg1137, Met1139, Leu1183 and Asn1185 in NSD2, and with Met1200, Met1221, Asn1267, Asn1197, Phe1198, and Thr1270 in NSD3. H3K27 (a.a. 23-29) peptide is stabilized by hydrogen bonds with Thr1995, Phe1997, Met1999, Arg2018, Met2020, Asn2021, Cys2063, Asn2066 and Thr2069 in NSD1, with Tyr1091, Asp1112, Thr1114, Phe1116, Thr1120, Arg1137, Met1139, Tyr1178, Asp1181, Leu1183, Asn1185 and Thr1188 in NSD2, and, with Thr1196, Phe1198, Met1200, Arg1219, Met1221, Asp1263, Cys1264, Leu1265, Asn1267, Arg1269 and Thr1270 in NSD3. H3K36 (a.a. 32-38) peptide is stabilized by hydrogen bonds with Asp1193, Phe1997, Met1999, Arg2018, Met2020, Asn2021, Glu2062 and Asn2066 in NSD1, with Thr1114, Phe1116, Arg1137, Met1139, Leu1180, Cys1182, Gly1184, Asn1185 and Glu1186 in NSD2, and, with Thr1270, Thr1196, Phe1198, Met1200, Arg1219, Met1221, Asp1263 and Cys1264 in NSD3. H3K79 (a.a. 75-81) peptide is stabilized by hydrogen bonds with Ile1994, Phe1997, Arg2018, Met2020, Thr2030, Lys2032, Cys2063, Asn2960, Asn2066 and Thr2069 in NSD1, with Trp1074, Ala1108, His1109, Ile1113, Thr1114, His1115, Phe1116, Phe1138, Met1139, Asn1140, Thr1149, Asn1179, Leu1180, Leu1183, Asn1185, and Arg1191 in NSD2, and with Asn1197, Phe1198, Met1200, Arg1219, Met1221, Thr1231, Lys1233, Tyr1260, Cys1264, Asn1267 and Thr1270 in NSD3. H4K20 (a.a. 16-22) peptide is stabilized by hydrogen bonds with Trp1955, Ile1994, Thr1995, Phe1997, Arg2018, Met2020, Tyr2059, Leu2061, Glu2062, Cys2063, Asn2066 and Thr2069 in NSD1, with Ala1108, Asn1111, Ile1113, Thr1114, Phe1116, Met1118, Arg1137, Met1139, Asn1140, Tyr1178, Asp1181, Cys1182, Asn1185 and Thr1188 in NSD2, and with Val1195, Thr1196, Asn1197, Phe1198, Arg1219, Met1221, Asn1222, Tyr1260, Asp1263, Asn1267 and Thr1270 in NSD3.

The docking of all histone peptides on the CD is associated with a significantly favorable docking energy, highlighting good substrate compatibilities. As a control experiment, we modelled the docking of a largely electronegative 7-mer poly-glutamic acid (polyE) peptide that induces significant steric hindrance in the peptide binding-pocket. The docking of the polyE peptide is associated with highly unfavorable binding energies, highlighting its poor fit to any of the NSD-CDs, as anticipated (Table 1).

Taken together, our data indicate that the histone-binding pocket of the NSD members is equally compatible with the histone peptides tested, offering steric and electrostatic matches for the stabilization of the H3 and/or H4 histone tail(s).

## Discussion

HMTases are essential in maintaining chromatin through modifications of histone tails. In this study, we examined the histone-lysine recognition and methylation properties of NSD1-CTD, NSD2-CTD, and NSD3-CTD *in vitro* to understand the extent of their catalytic properties and provide medicinal chemistry insights into the design of NSD inhibitors.

Some HMTases, such as SET9, do not retain significant methyltransferase activity *in vitro* [50], unlike CTDs of NSD1, NSD2, and NSD3 (Figure 2). Although significant differences in methyltransferase activities have been observed, it is unambiguous that the CTD can recognize and methylate various histone substrates *in vitro* (Figure 2). Our data indicate that the CTDs of NSD1, NSD2, and NSD3 recognize and methylate H3K4, H3K9, H3K27, H3K36, H3K79 and H4K20 *in vitro* (Figure 2). In addition, other work has shown H4K44 methylation by the NSD2-SET domain on octamers [36]. This contrasts with *in vivo* results where the NSDs are exclusive to one substrate H3K36 or H4K20.

*In vivo,* NSD2 activity is specific to either H3K36 or H4K20 depending on the cellular context [17,20]. The level of HMTase activity also depends on the nature of the substrate (peptide, histone, or nucleosome). The NSD2 SET domain alone can also methylate H3K36 *in vitro*, generating at least H3K36me1 [36]. H3K36 methylations have been predominantly reported for the NSD members and have significant roles in transcriptional activation, alternative splicing events, and DNA repair [24,54-56]. Methylations on H3K4, H3K9, H3K27, H3K79, and H4K20 by NSD members have also been identified. NSD members might possibly have unique methylation capabilities that respond to a specific cellular context *in vivo.*

The methylation of non-histone protein targets has previously been shown for HMTases such as G9a targets, WIZ, CDYL1, and ACINUS [57], and SET7/9 target, SUV39H1 [56]. G9a is capable of automethylation on the non-enzymatic N-terminal part of the enzyme [57]. Interestingly, NSD1 and NSD2 methylate both histone and non-histone substrates equally. NF-κB is activated through NSD1-mediated mono- and di-methylation on lysine 218 [42]. NSD2 directly interacts with NF-κB for the activation of target genes, including IL-6, IL-8, VEGFA, cyclin D, Bcl-2, and survivin in castration-resistant prostate cancer cells (CRPC) [4]. NSD2 is recruited to the target gene promoters upon induction and mediates NF-κB activation-associated elevation of histone H3K36me2 and -me3 marks at promoters [8,42]. Less is known about NSD3, but non-histone methylation is potentially one of its biological functions as well.

In the absence of substrate, the histone-binding pocket of the NSD members is closed, similar to the H3K9 HMTase MLL1 [53]. Our data suggest that the binding of the histone-peptide itself is not entirely responsible for displacing the post-SET regulatory loop *in vivo*. Instead, the binding of histones or nucleosomal DNA could be an allosteric effector that triggers the opening of the catalytic domain. Nonetheless, our assays indicate that, *in vitro*, the binding of either H3 or H4 histone triggers the opening of NSD-CD. The binding cavity appears to equally accommodate the 7-mer histone peptides tested (H3K4 a.a. 1-7; H3K9 a.a. 5-11; H3K27 a.a. 23-29; H3K36 a.a. 32-38; H3K79 a.a. 75-81; H4K20 a.a. 16-22), likely due to all being electrostatically positively charged and presenting homologous steric hindrance. In the case of NSD2, the binding of the H4K20 peptide is favorable and stabilized by an extensive network of hydrogen bonds. The binding of the H3K36 peptide is strongly anchored in the opened-SET domain of NSD2 with ten hydrogen bonds (thirteen for H4K20 a.a. 16-22) (Figure 5). However, a better catalytic efficiency *in vitro* is observed on H3K36 rather than H4K20, which could perhaps be ascribed to a more favorable conformation of the lysine substrate (Figure 2). Differences calculated in binding energies can be due to differential stabilization properties, which are not necessarily linked with catalytic efficiencies.

Analysis of peptide-protein interactions reveals a conserved binding motif for all the NSD members composed of a quartet of Phenylalanine, Arginine, Methionine and Asparagine (Figures 4 and 5). The trio of conserved Phenylalanine, Arginine and Methionine is located on the pre-SET domain Phe1197 (NSD1), Phe1116 (NSD2), Phe1198 (NSD3); Arg2018 (NSD1), Arg1137 (NSD2), Arg1219 (NSD3); Met2020 (NSD1), Met1139 (NSD2), Met1221 (NSD3) whereas the Asparagine consensus Asn2066 (NSD1), Asn1185 (NSD2) and Asn1267 (NSD3) sits at the interface of the SET and post-SET region, on the flexible regulatory loop we previously identified [9]. In addition, a specific conserved binding motif can be identified for only a pair of NSDs: Met1999 (NSD1) and Met1200 (NSD3); Tyr2059 (NSD1) and Tyr1178 (NSD2); Thr114 (NSD2) and Thr1196 (NSD3). Moreover, peptide-protein interaction specific for each NSD completes the binding motif with Cys2063 (NSD1 specific), Asn1140 (NSD2 specific), Asn1197 & Asp1263 & Thr1270 (NSD3 specific). Taken together, the differences observed within the binding motifs amongst the NSDs offer possibilities of discrimination that may be exploited in a drug design strategy targeting the histone tail-binding site.

The post-SET regulatory loop sits on top of the histone-peptide, stabilizing the complex and forcing the lysine substrate into the catalytic channel extending deep toward the co-factor-binding site. In all cases, the lysine substrate extends into the narrow channel connecting the co-factor-binding site in order to react and is anchored by two hydrogen bonds, except for NSD3 with H3K4 and H3K9 where the lysine substrate is bonded by a single hydrogen bond. This double-hydrogen bond is found in other lysine-HMTases and appears to be associated with catalytic efficiency. This observation is consistent with the peptide-bound structures of H3K9-HMTases (GLP and Dim-5) where the Lys9 is stabilized by a pair of analogous hydrogen bonds [52]. A similar conserved double-hydrogen bond is observed in the structures of H4K20-SETD8, H3K4-SETD7, and H3K27-vSET [52]. In NSD2, the H3K36 lysine is anchored by two hydrogen bonds whereas the H4K20 lysine is strongly held in place by three hydrogen bonds. In addition, the H3K36 a.a. 32-38 peptide is stabilized by a network of ten hydrogen bonds compared to thirteen for the H4K20 a.a. 16-22 peptide (Figure 5). This observation is consistent with a more favorable binding energy of H4K20 a.a. 16-22 compared to the H3K36 a.a. 32-38 peptide (Table 1). Our data indicate that NSD3-CTD has comparably poor HMTase activities on H3K4 and H3K9, which appear to be correlated with the Lys4 and Lys9 being anchored by only one hydrogen bond. Overall, the stabilization of the lysine substrate is established by a pair of hydrogen bonds that tightly anchor the lysine substrate in the narrow catalytic channel, affecting catalytic efficiency (Figures 2, 3, 4 and 5).

## Conclusions

Our data suggest that the NSD members may not be exclusive to a single histone mark *in vivo*. We show that the CTDs of NSD members can carry out pan-methylation of lysine substrates and generate K-me1, K-me2, and K-me3 species *in vitro*. However, *in vivo* cell-based assays have predominantly identified K-me2 or K-me3 species depending on the cellular context [2,13,17,18]. Our molecular modelling data indicate that the binding-groove can equally accommodate K-me2 or K-me3 species. This is consistent with a regulatory mechanism distinct from the CD and carried out by the PHD4 domain to control the localization and the degree of methylation by NSD members [46]. Interestingly, the PWWP domain of the fission yeast Set9 partner, Pdp1, controls Set9-mediated H4K20 methylation and appears to directly interact with histones for Set9 localization [50]. Moreover, the sequence conservation is high between Pdp1-PWWP and both PWWP domains of NSD1, NSD2, and NSD3 [50]. The hydrophobic residues in Pdp1-PWWP responsible for the recognition of H4K20 marks are also conserved in the two NSD-PWWP domains [50]. Like Pdp1, the NSD members may localize to chromatin through the assistance of PWWP domains. Further analysis to elucidate the role of the PWWP domains in the NSD members will greatly improve our understanding of the regulation of histone-lysine methylations.

## Additional file

Additional file 1
**Standard plots of the total NAMD energy** ***versus*** **timestep during MD simulations.** The total energy in kcal/mol of the system during the MD simulations is plotted *versus* time. The plots provide an overview of the five cycles of energy minimization and molecular dynamic simulations.

## Abbreviations

NSD: Nuclear receptor binding SET domain
MMSET: Multiple myeloma SET domain
HMTase: Histone lysine Methyltransferase
SAM: S-adenosylmethionine
MD: Molecular dynamics
RMSD: Root-mean-square deviation
