# Supplementary material for: In vitro histone lysine methylation by NSD1, NSD2/MMSET/WHSC1 and NSD3/WHSC1L
Source: BMC Struct Biol. 2014 Dec 12;14:25. doi: 10.1186/s12900-014-0025-x (PMC4280037; doi:10.1186/s12900-014-0025-x)
Supplement: Additional file 1 — Standard plots of the total NAMD energy versus timestep during MD simulations. The total energy in kcal/mol of the system during the MD simulations is plotted versus time. The plots provide an overview of the five cycles of energy minimization and molecular dynamic simulations. [file 12900_2014_25_MOESM1_ESM.pdf]

in vitro Histone Lysine Methylation by NSD1, NSD2/MMSET/WHSC1, and NSD3/WHSC1L

Masayo Morishita[1], Damiaan Mevius[1], Eric di Luccio[1]

[1]Kyungpook National University, School of Applied Biosciences,
Daegu, 702-701 - South Korea

Figure Legend for the supplementary data

Fig. S1 – Standard plots of the total NAMD energy versus timestep during MD simulations

The total energy in Kcal/mol of the system during the MD simulations is plotted *versus* time. The plots provide an overview of the five cycles of energy minimization and molecular dynamic simulations.

Fig. S1

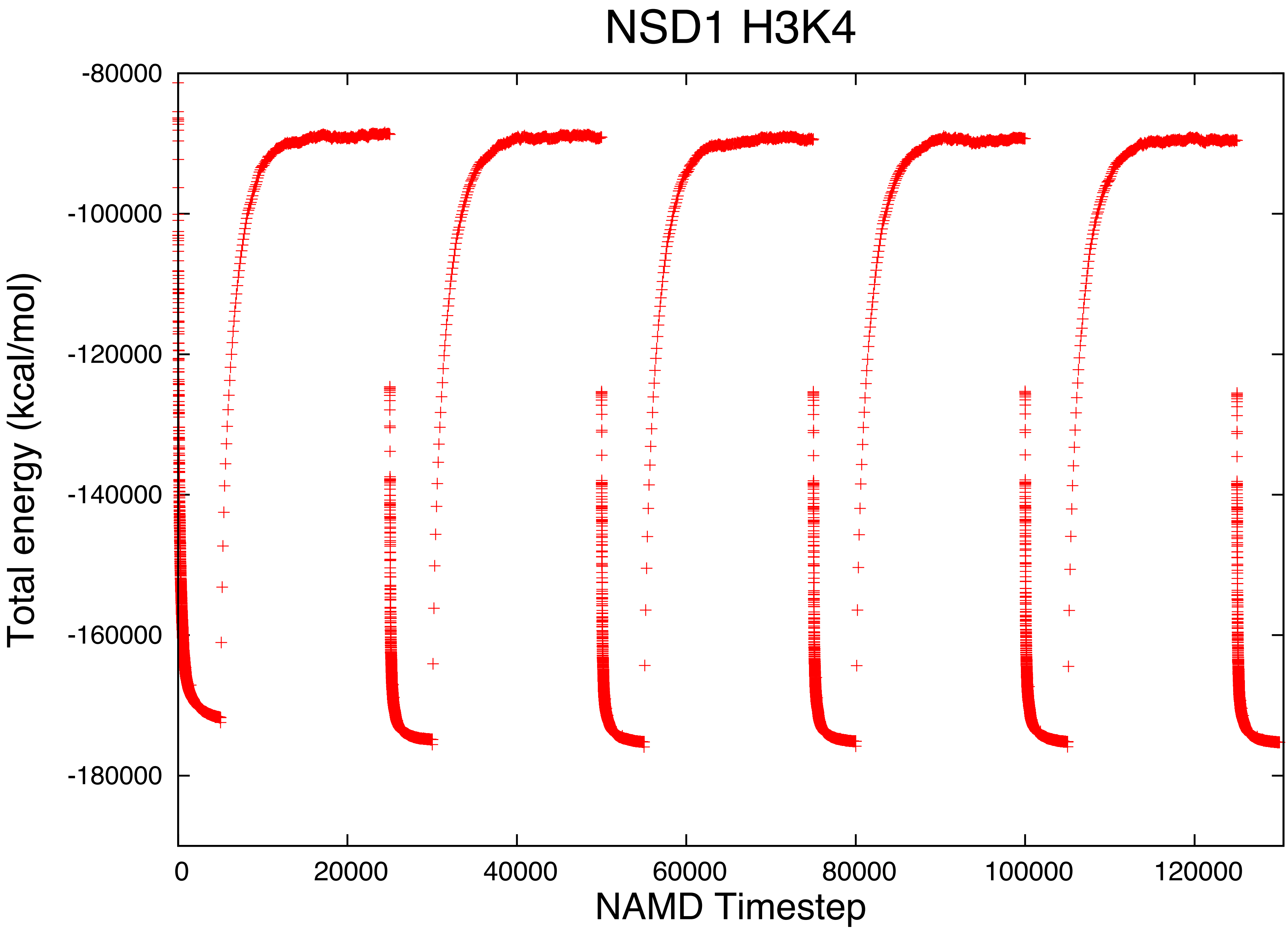
NSD1 H3K4
Total energy (kcal/mol)
-80000
-100000
-120000
-140000
-160000
-180000
0
20000
40000
60000
80000
100000
120000
NAMD Timestep

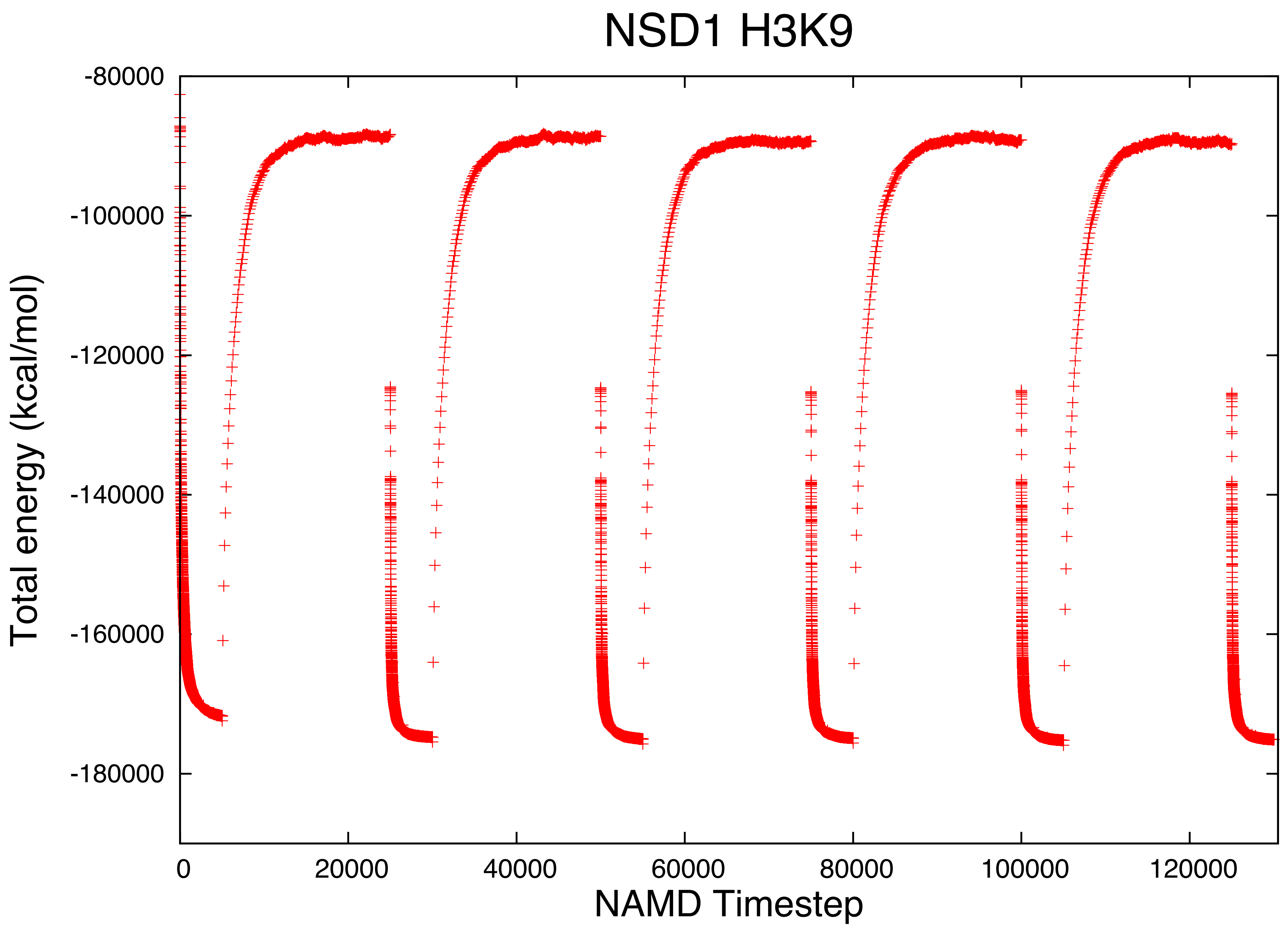
NSD1 H3K9
Total energy (kcal/mol)
-80000
-100000
-120000
-140000
-160000
-180000
0
20000
40000
60000
80000
100000
120000
NAMD Timestep

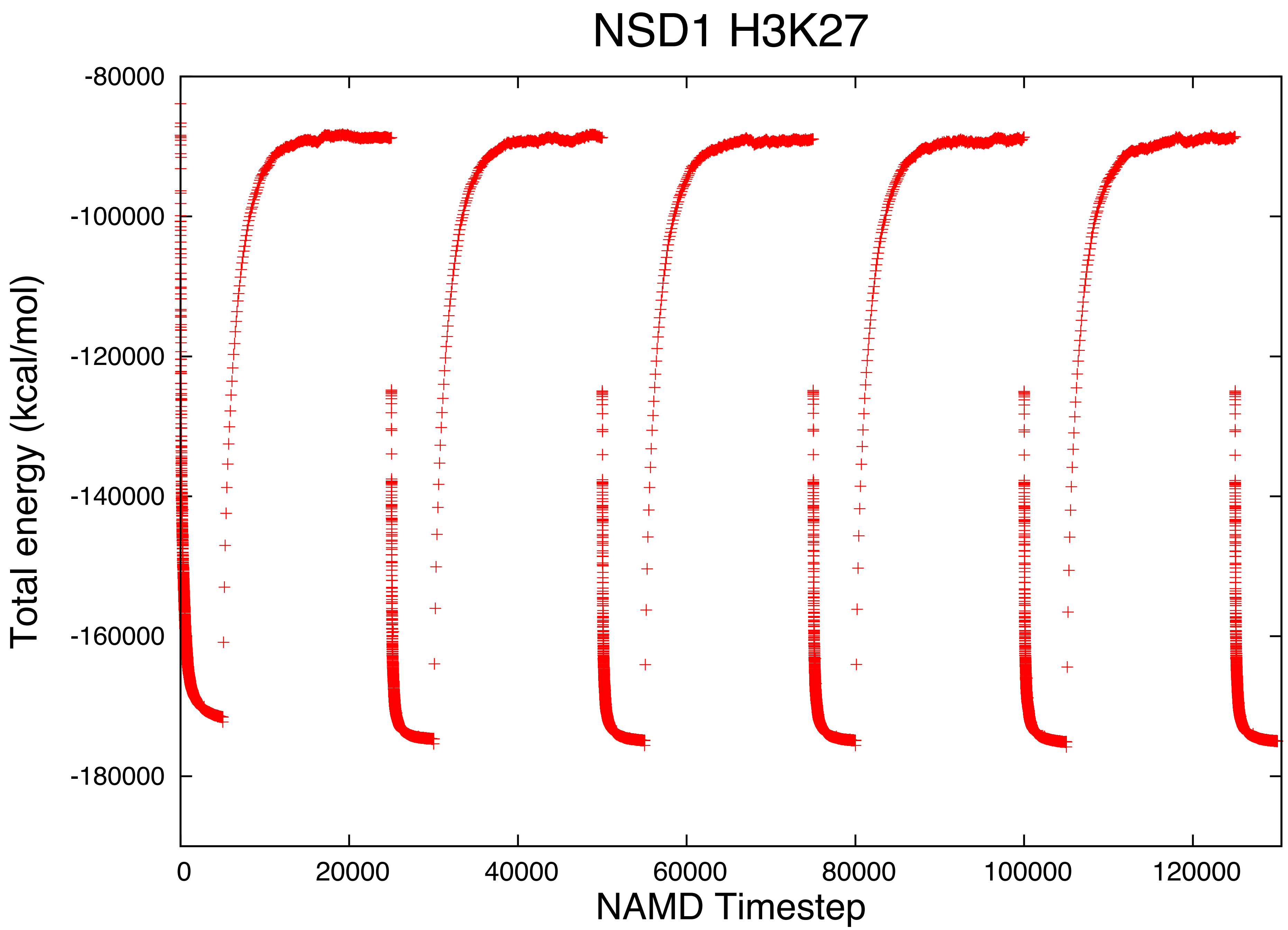
NSD1 H3K27
Total energy (kcal/mol)
-80000
-100000
-120000
-140000
-160000
-180000
0
20000
40000
60000
80000
100000
120000
NAMD Timestep

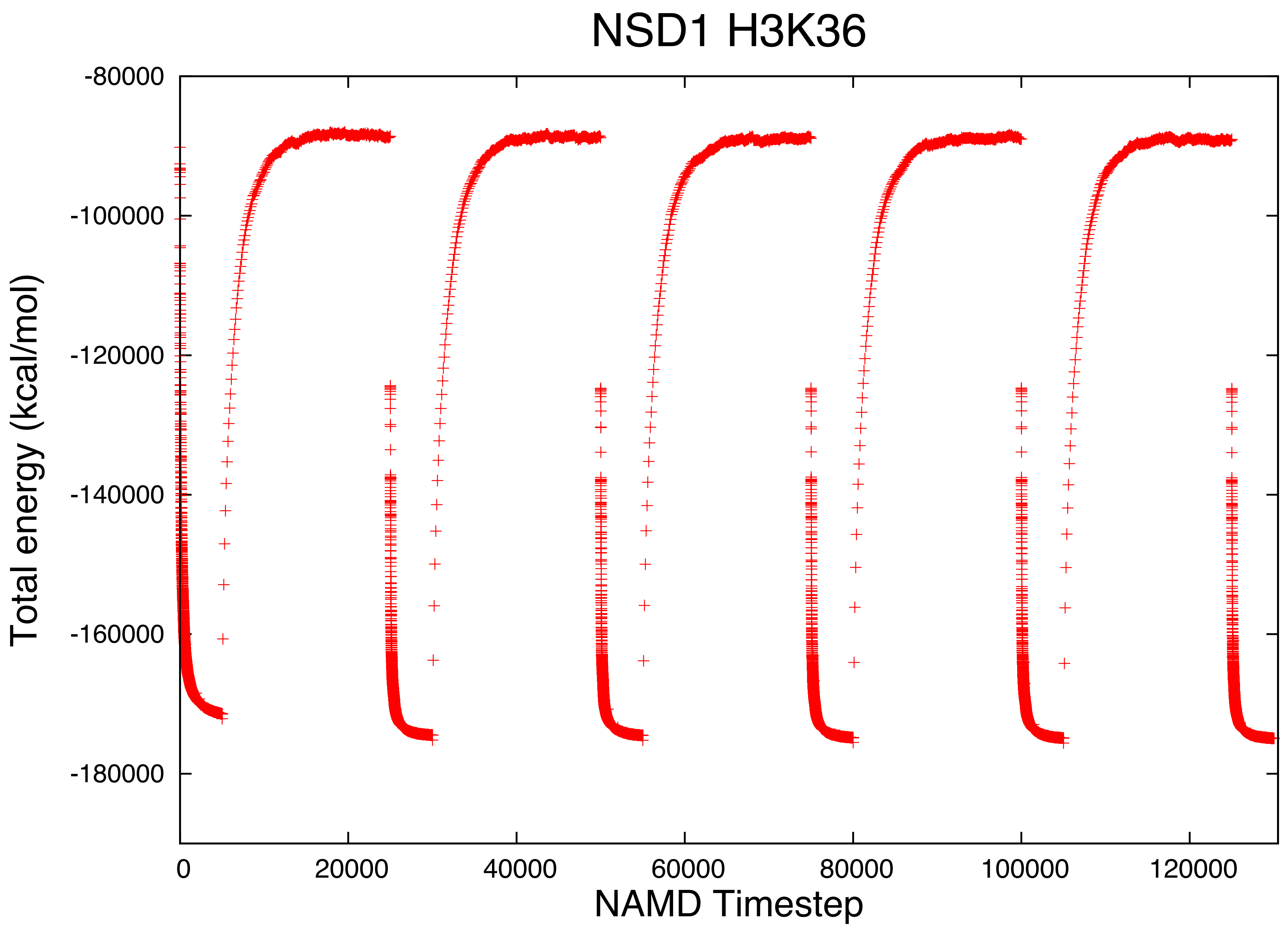
NSD1 H3K36
Total energy (kcal/mol)
-80000
-100000
-120000
-140000
-160000
-180000
0
20000
40000
60000
80000
100000
120000
NAMD Timestep

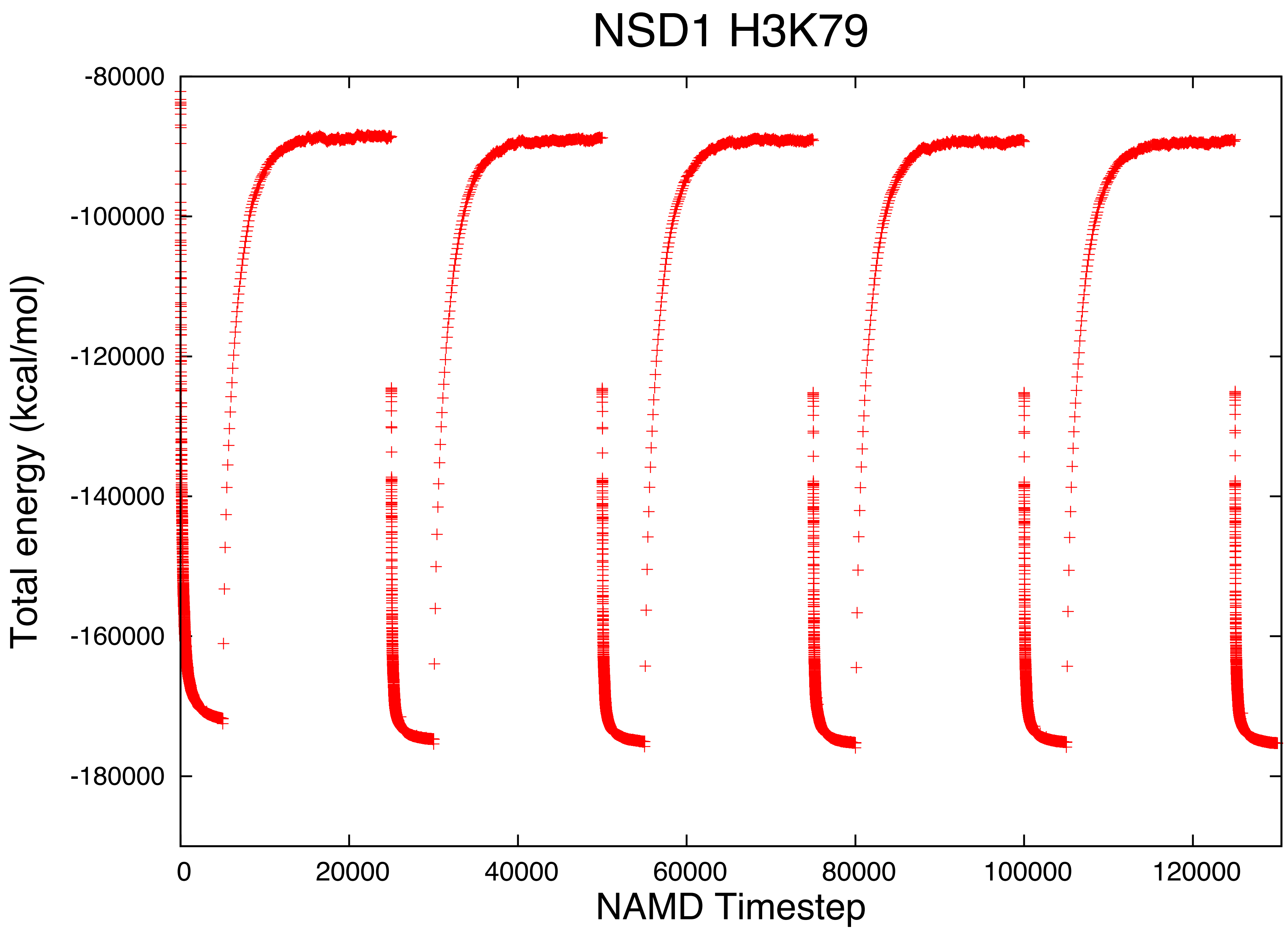
NSD1 H3K79
Total energy (kcal/mol)
NAMD Timestep
-80000
-100000
-120000
-140000
-160000
-180000
0
20000
40000
60000
80000
100000
120000

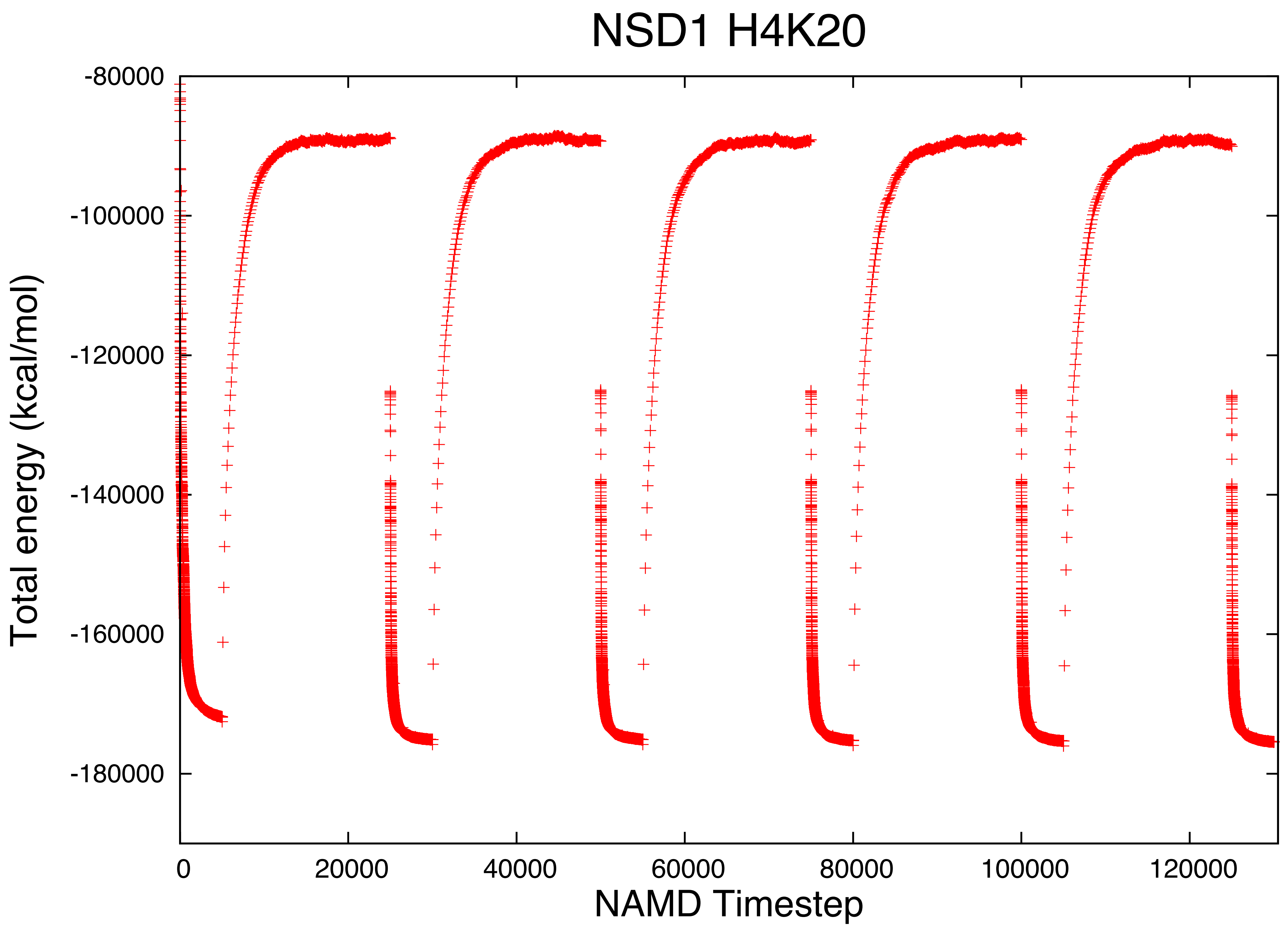
NSD1 H4K20
Total energy (kcal/mol)
-80000
-100000
-120000
-140000
-160000
-180000
0
20000
40000
60000
80000
100000
120000
NAMD Timestep

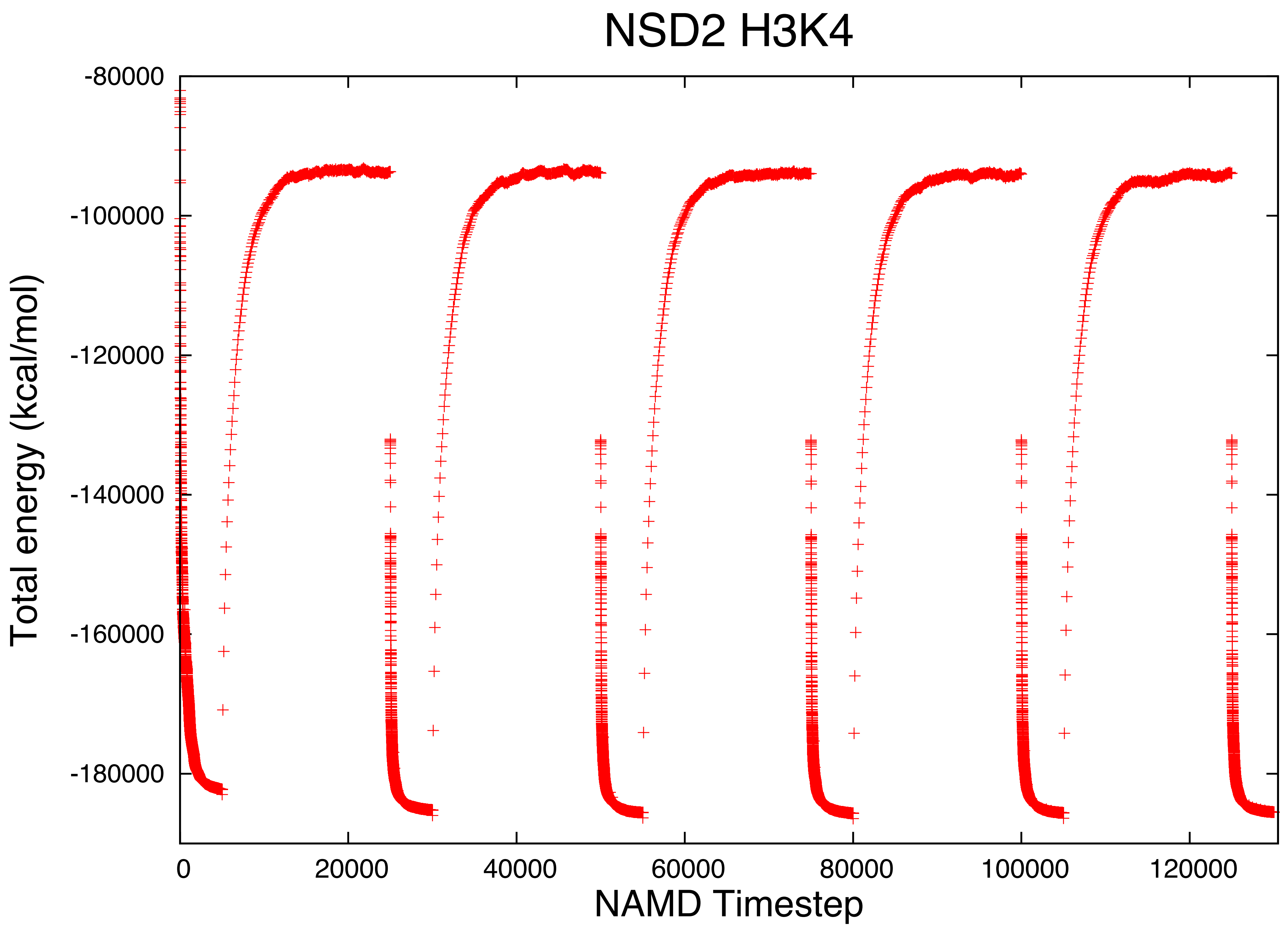
NSD2 H3K4
-80000
-100000
-120000
-140000
-160000
-180000
Total energy (kcal/mol)
0
20000
40000
60000
80000
100000
120000
NAMD Timestep

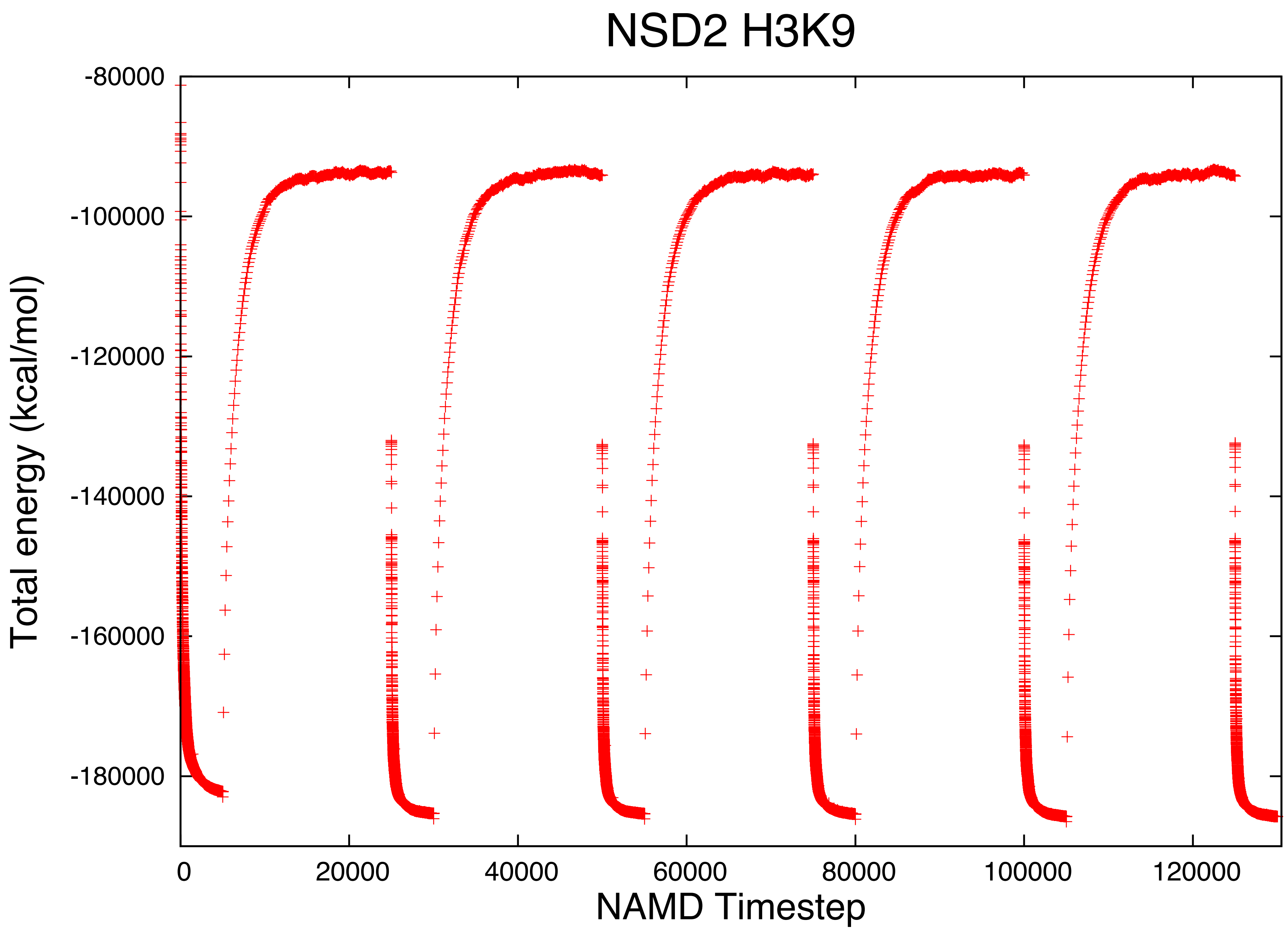
NSD2 H3K9
Total energy (kcal/mol)
-80000
-100000
-120000
-140000
-160000
-180000
0
20000
40000
60000
80000
100000
120000
NAMD Timestep

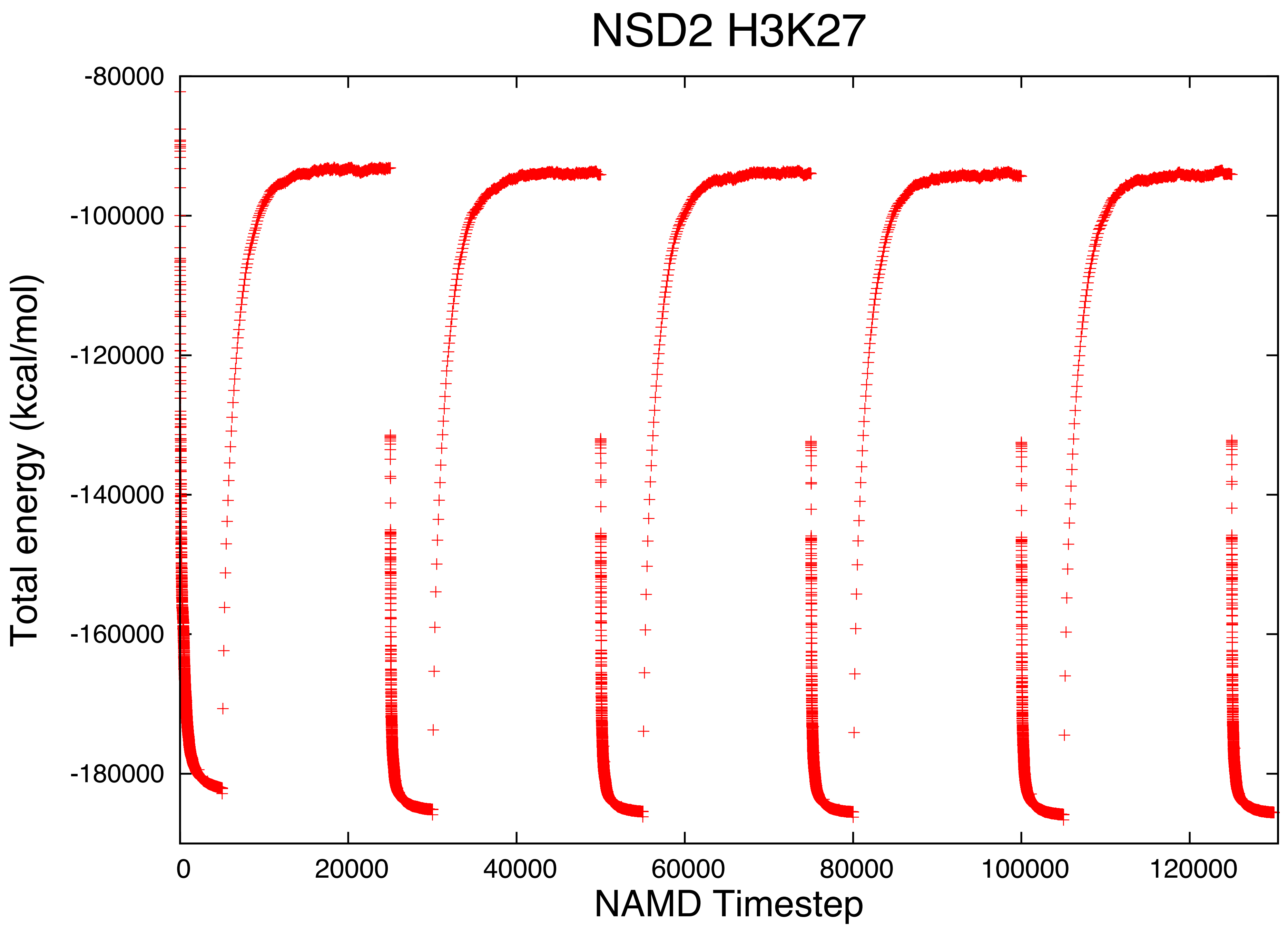
NSD2 H3K27
-80000
-100000
-120000
-140000
-160000
-180000
Total energy (kcal/mol)
0
20000
40000
60000
80000
100000
120000
NAMD Timestep

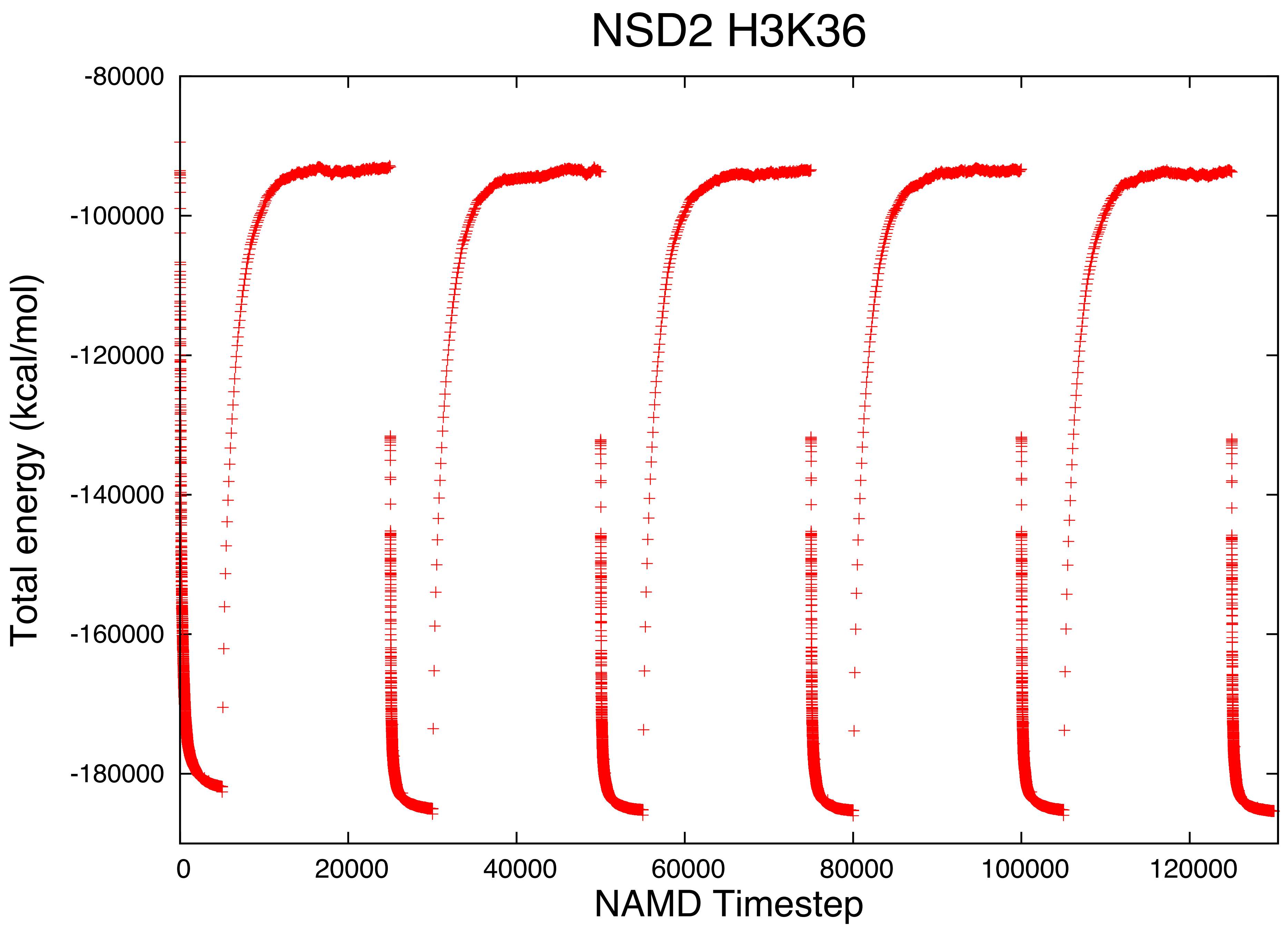

NSD2 H3K36
Total energy (kcal/mol)
NAMD Timestep
-80000
-100000
-120000
-140000
-160000
-180000
0
20000
40000
60000
80000
100000
120000

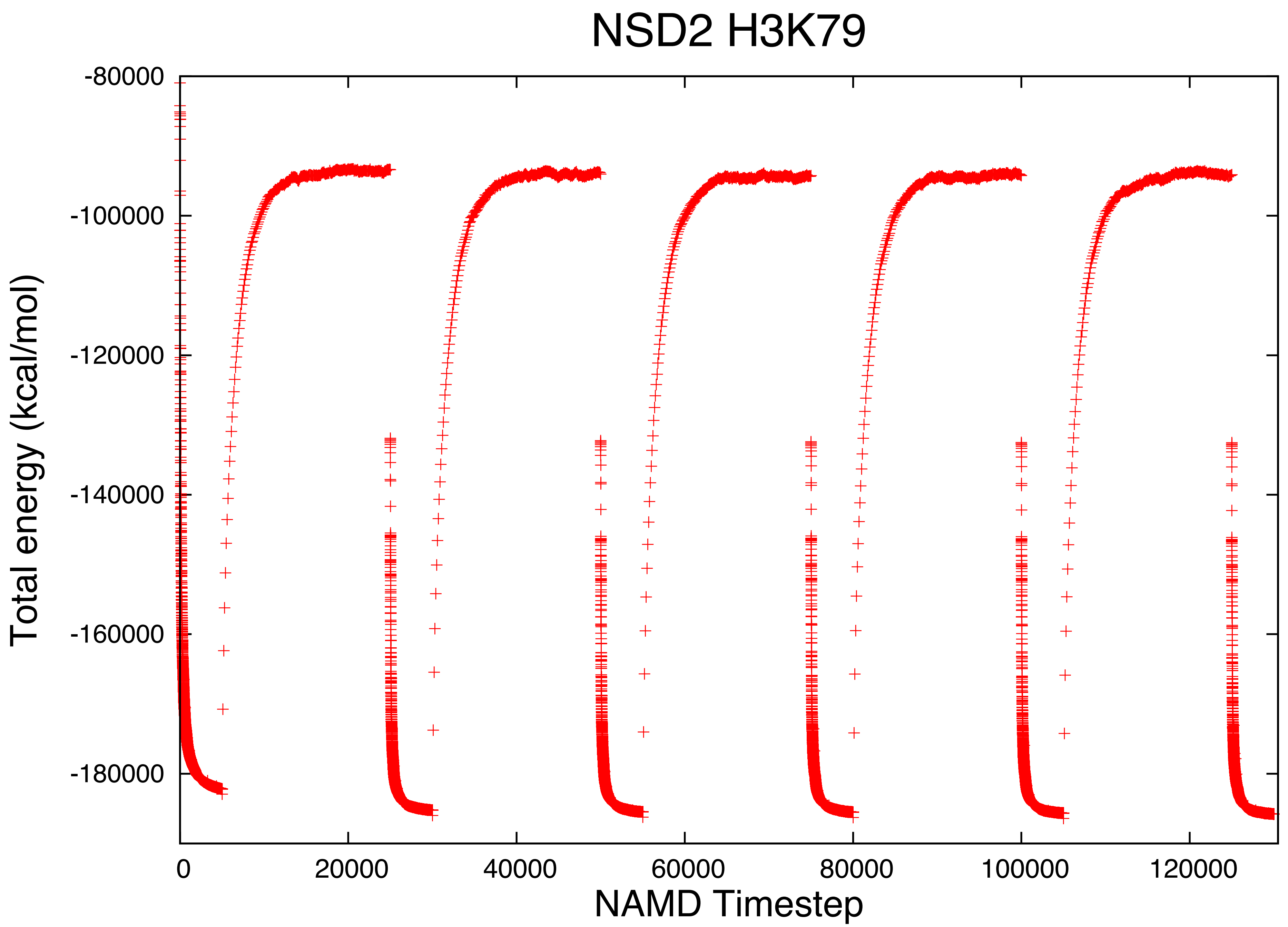

NSD2 H3K79
-80000
-100000
-120000
-140000
-160000
-180000
Total energy (kcal/mol)
0
20000
40000
60000
80000
100000
120000
NAMD Timestep

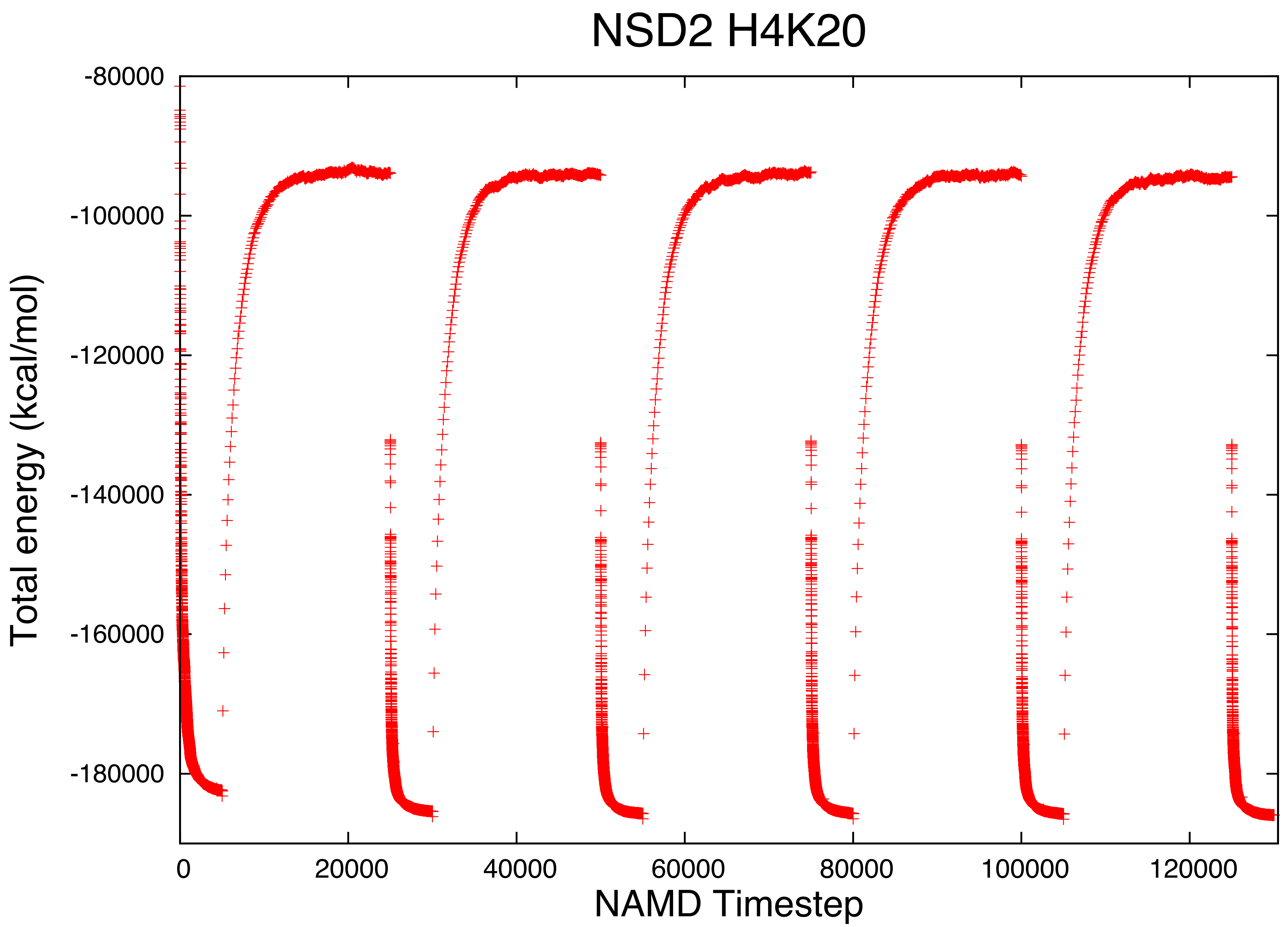
NSD2 H4K20
Total energy (kcal/mol)
-80000
-100000
-120000
-140000
-160000
-180000
NAMD Timestep
0
20000
40000
60000
80000
100000
120000

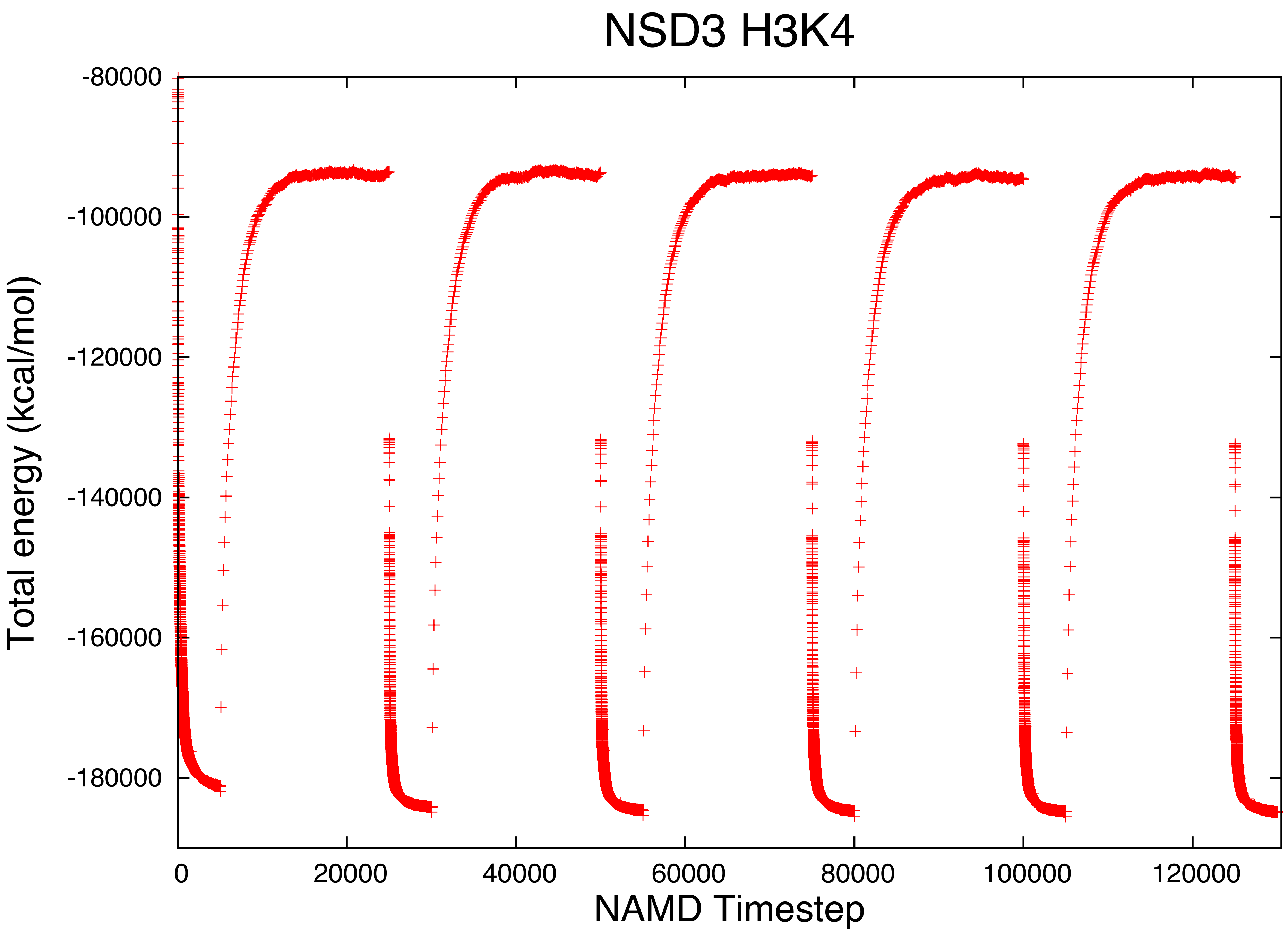
NSD3 H3K4
Total energy (kcal/mol)
-80000
-100000
-120000
-140000
-160000
-180000
0
20000
40000
60000
80000
100000
120000
NAMD Timestep

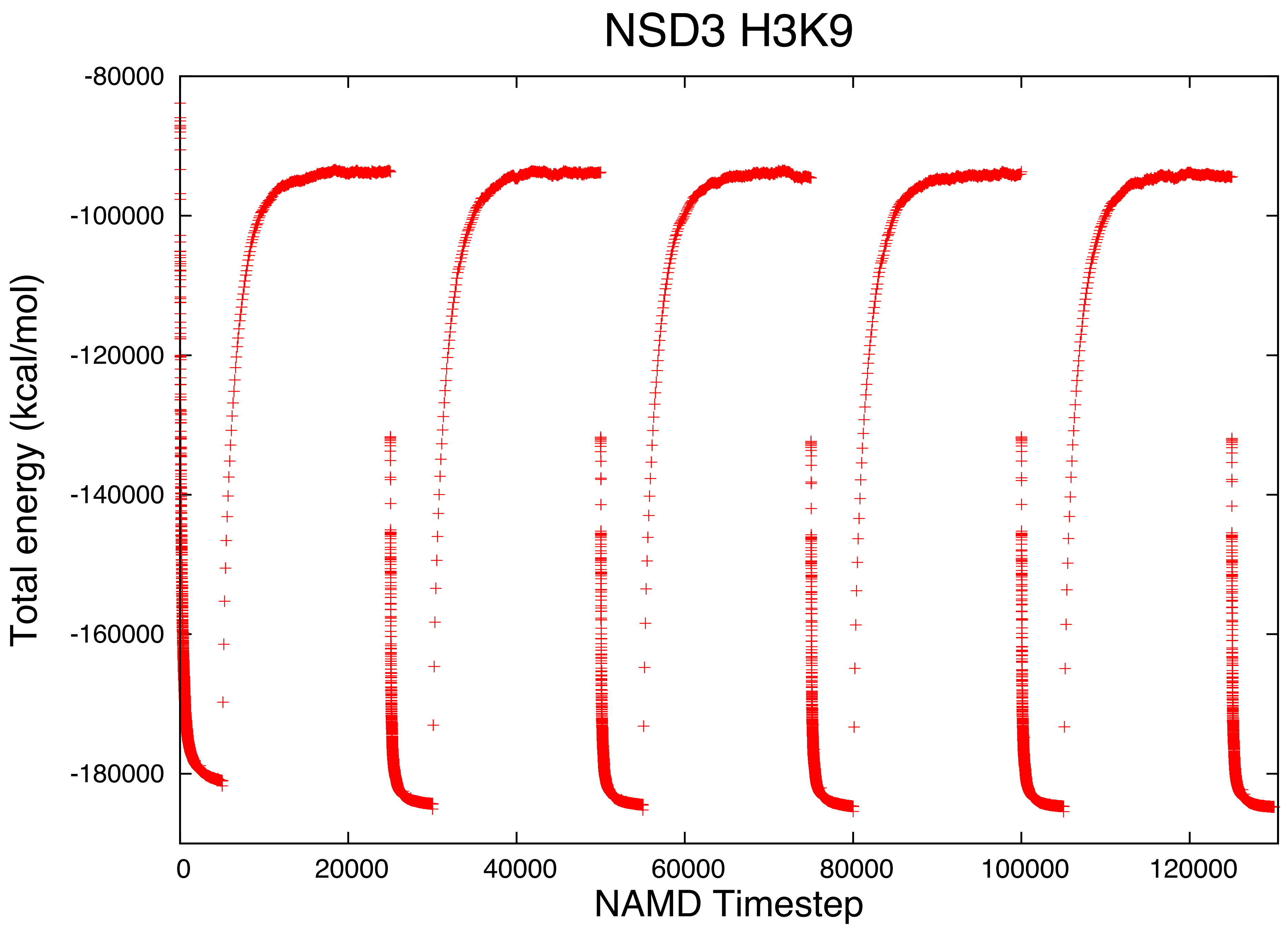
NSD3 H3K9
Total energy (kcal/mol)
NAMD Timestep
-80000
-100000
-120000
-140000
-160000
-180000
0
20000
40000
60000
80000
100000
120000

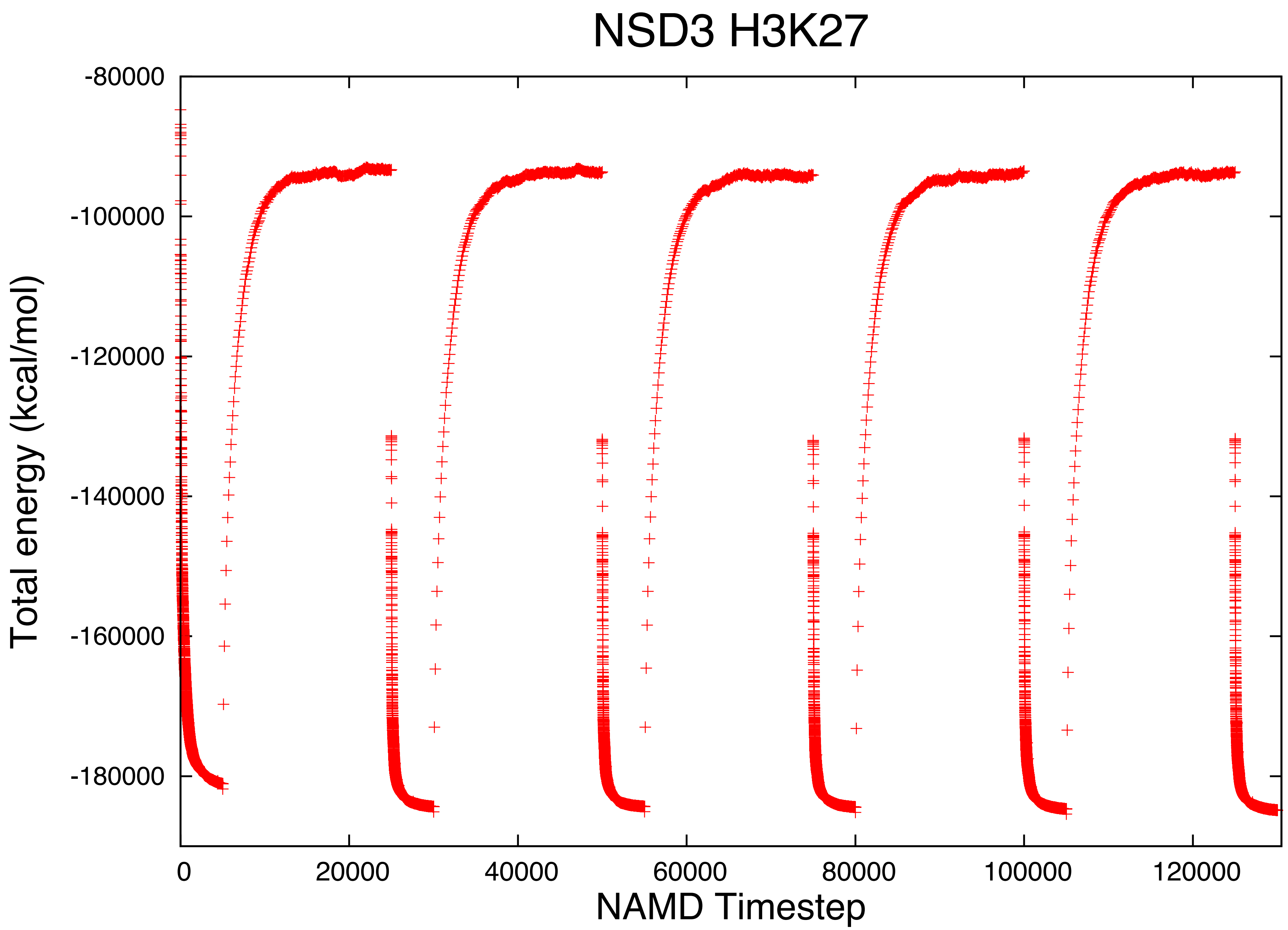
NSD3 H3K27
Total energy (kcal/mol)
NAMD Timestep
-80000
-100000
-120000
-140000
-160000
-180000
0
20000
40000
60000
80000
100000
120000

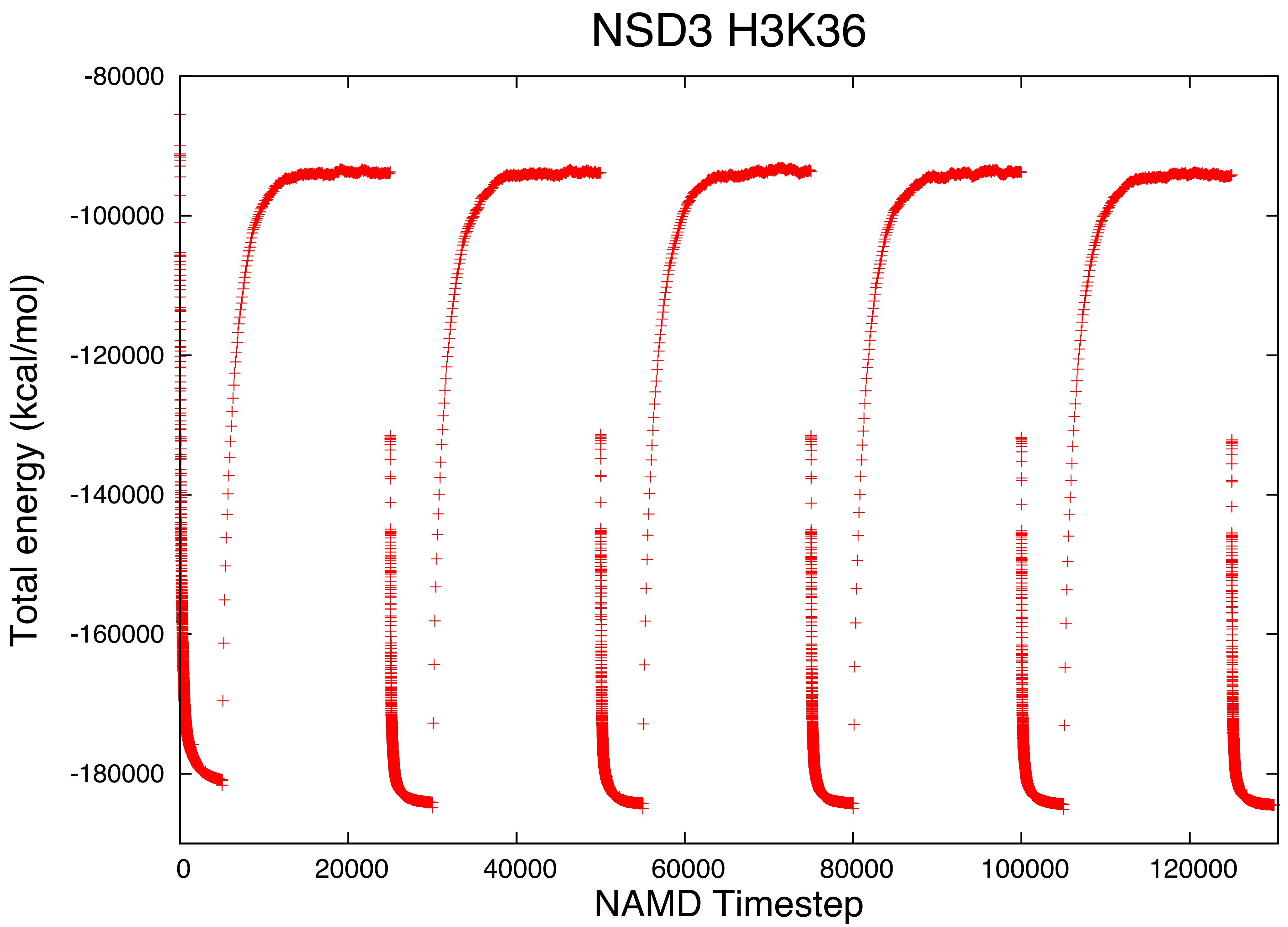

NSD3 H3K36
Total energy (kcal/mol)
-80000
-100000
-120000
-140000
-160000
-180000
0
20000
40000
60000
80000
100000
120000
NAMD Timestep

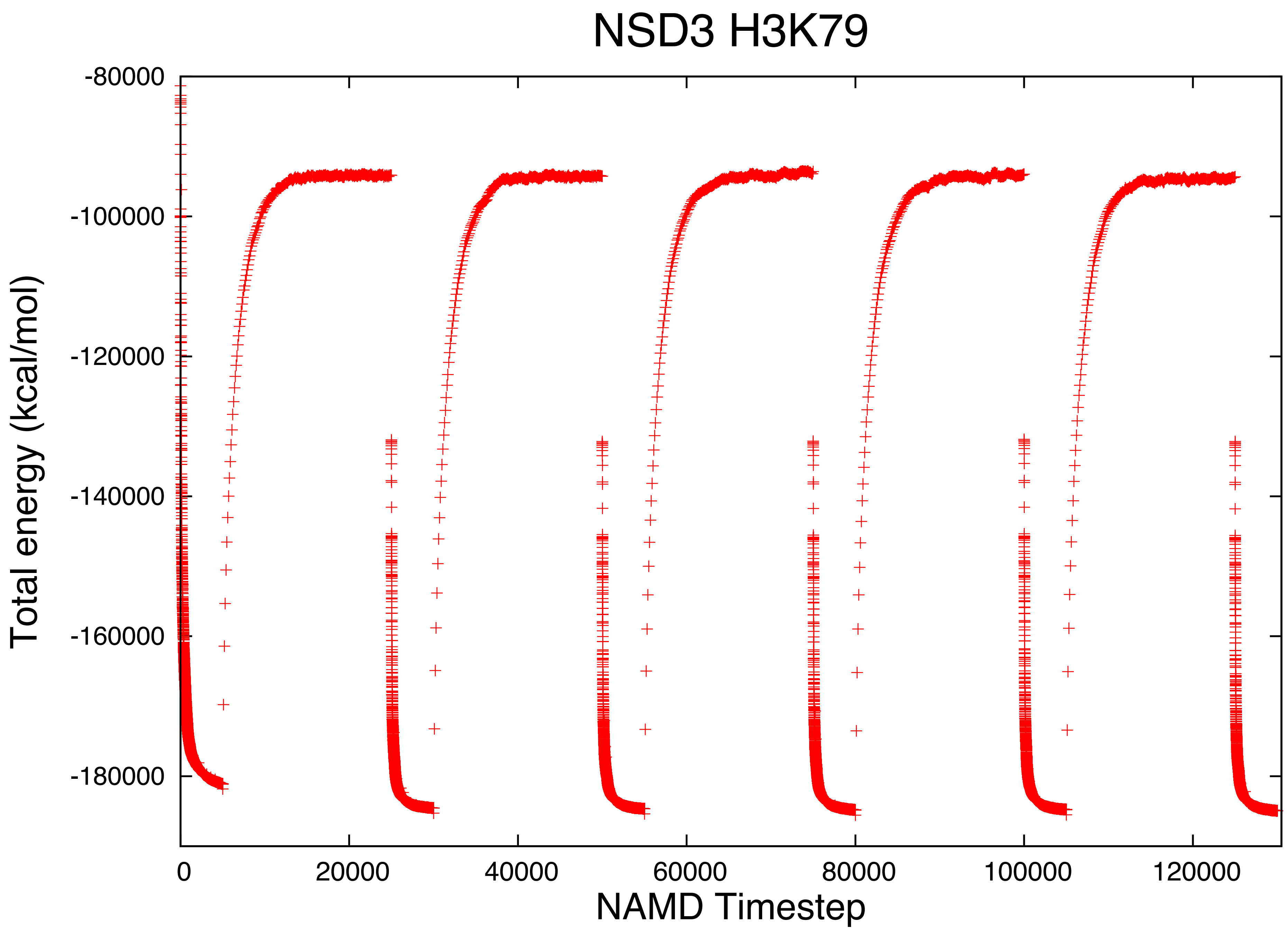
NSD3 H3K79
Total energy (kcal/mol)
NAMD Timestep
-80000
-100000
-120000
-140000
-160000
-180000
0
20000
40000
60000
80000
100000
120000

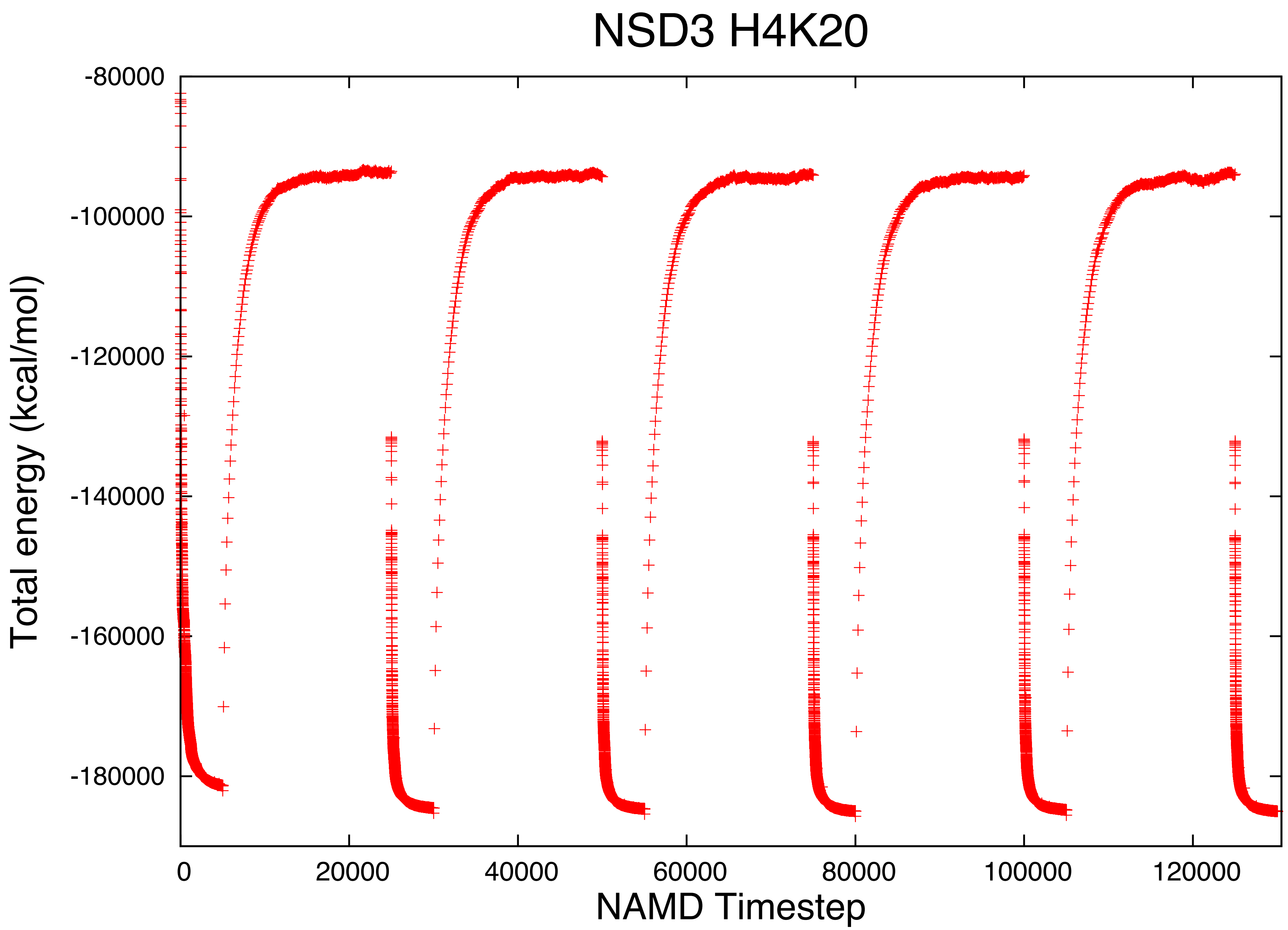

NSD3 H4K20
Total energy (kcal/mol)
-80000
-100000
-120000
-140000
-160000
-180000
0
20000
40000
60000
80000
100000
120000
NAMD Timestep
